# Development and validation of the childhood allergy comprehensive screening questionnaire and Its application in a cross-sectional survey of allergic diseases among children aged 0–6 Years in Shanghai, China

**DOI:** 10.3389/fpubh.2026.1906537

**Published:** 2026-07-29

**Authors:** Jinshan Yang, Nina Ma, Fang Tian, Aiqin Gu, Changfei Shi, Shaoye Huo, Yuhuan Wei, Yunhua Zhao, Lihong Wang, Yun Li, Yuan Cui, Chunhai Shao

**Affiliations:** 1 Department of Nutrition, Shanghai Fifth People's Hospital, Fudan University, Shanghai, China; 2 Center of Community-Based Health Research, Fudan University, Shanghai, China; 3 Department of Nutrition, Huashan Hospital, Fudan University, Shanghai, China; 4 Gumei Community Health Service Center, Shanghai, China; 5 Department of Epidemiology, School of Public Health, Fudan University, Shanghai, China; 6 Department of Child Health Care, Shanghai Minhang Maternal and Child Health Care Hospital, Shanghai, China; 7 Department of Dermatology, Huashan Hospital Research Center of Allergy and Disease, Fudan University, Shanghai, China

**Keywords:** allergic asthma, allergic rhinitis, atopic dermatitis, children, food allergy, screening

## Abstract

**Introduction:**

Childhood allergic diseases constitute a substantial global health burden, with children aged 0–6 years representing a highly vulnerable population during early immunological transition. Evidence regarding the associations of lifestyle, household environments, and prenatal exposures with these conditions remains inconsistent and heterogeneous, and epidemiological data for this critical age group are still limited. This study aimed to develop and validate a standardized screening instrument and to evaluate these potential associations.

**Methods:**

This study utilized a two-phase design: Phase I (Clinical Validation) evaluated the psychometric properties of a newly developed, culturally localized 24-item Childhood Allergy and Asthma Comprehensive Screening Questionnaire (CACSQ) against clinical gold-standard physician diagnoses in a hospital-based case-control study comprising 98 cases and 98 matched controls. Phase II (Epidemiological Survey) applied the validated CACSQ in a district-wide cross-sectional investigation. Using multi-stage stratified random cluster sampling, 3,064 children aged 0–6 years were enrolled in Minhang District, Shanghai, China. Multivariable logistic regression models were employed to identify independent perinatal, residential-environmental, and familial factors associated with childhood allergies.

**Results:**

The CACSQ demonstrated high accuracy, with an AUC of 0.88 (*P*<*0.001*), sensitivity of 92%, specificity of 76%, and Kappa coefficient of 0.698. Among 3,064 children aged 0–6 years, the overall CACSQ-identified prevalence of food allergy, asthma, atopic dermatitis, and allergic rhinitis was 14.7%, 8.4%, 18.9%, and 20.0%, respectively. Male sex, higher socioeconomic status, only-child status, history of antibiotic use and pneumonia, and family history of allergy were associated with elevated risk of allergic diseases. In contrast, sleep duration ≥ 12 h/day and window-opening ventilation were associated with lower odds of allergic diseases. Age effects varied by condition; the odds of atopic dermatitis decreased with age, whereas the odds of allergic rhinitis increased.

**Conclusion:**

Genetic predisposition and modifiable environmental/lifestyle factors significantly associated with early childhood allergies. The validated CACSQ offers a reliable, low-cost screening tool for early detection and future longitudinal cohort studies.

## 1 Introduction

Allergic diseases, including asthma, allergic rhinitis (AR) atopic dermatitis (AD), and food allergies (FA), constitute a major global public health challenge with a steadily increasing prevalence over the past few decades (1). These conditions represent a significant source of morbidity, impairing quality of life, sleep, and cognitive development, and imposing a substantial economic burden on healthcare systems and families worldwide (2–5). The rising trend in pediatric allergic diseases is particularly evident in China, where they remain a pressing concern. Recent multicenter studies reveal a high and age-dependent burden: among children aged 6–11 years, the prevalence of FA is 11% (6), while the diagnosis rates of AR, AD, and asthma in children aged 6–18 years are 14.2%, 11.4%, and 9.2%, respectively (7). More strikingly, among younger children aged 3–14 years, current prevalence rates escalate dramatically to 28.8% for AR, 38.8% for AD, and 20.2% for asthma (8).

This gradient underscores early childhood as a period of exceptional vulnerability, a notion strongly supported by a specialized database survey in Shanghai which found that over 80% of pediatric allergy cases have their onset in the 0–6–year age group, with half of all initial diagnoses occurring in preschool children (3–6 years) (9). Despite the recognized vulnerability of early childhood, epidemiological data specifically for the 0–6 years age group in China remain scattered, hindering targeted intervention. To address these problems, we conducted a population-based cross-sectional study to determine the current prevalence and influencing factors of allergic diseases among children aged 0–6 years in China. Furthermore, we developed and implemented a standardized screening questionnaire to ensure rigorous and consistent case definition across this pivotal pediatric age group.

## 2 Methods

### 2.1 Study design

**Figure 1:**
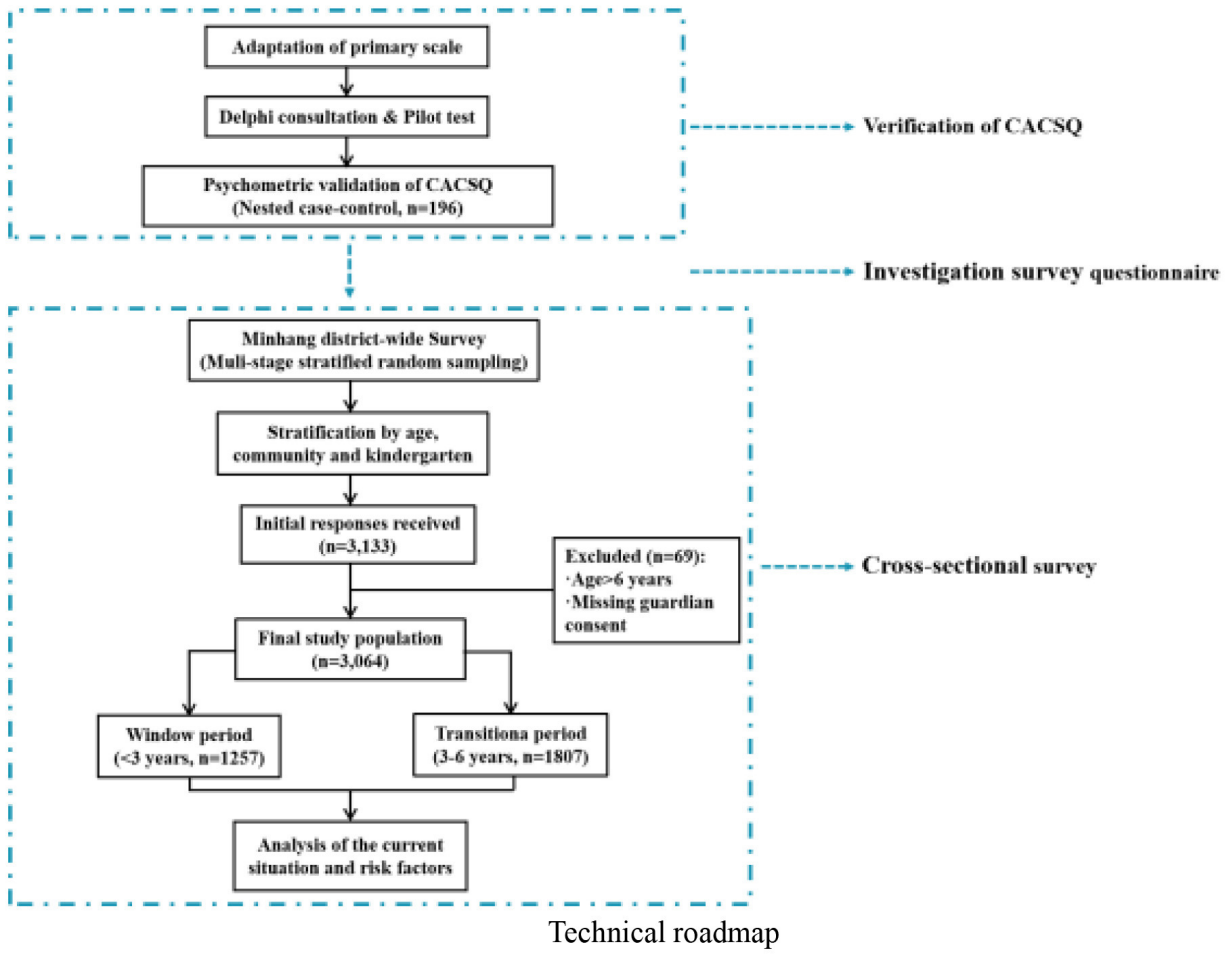
Technical roadmap.

This work integrates a hospital-based validation study (phase I) and a subsequent district-wide cross-sectional survey (phase II), Figure 1. The studies involving humans were approved by Medical Ethics Committee of Shanghai Fifth People's Hospital Affiliated to Fudan University (2024177). Written informed consent was obtained from the parents or legal guardians of all participating children prior to enrollment.

### 2.2 Phase I: development and validation of CACSQ

The Childhood Allergy and Asthma Comprehensive Screening Questionnaire (CACSQ) was developed through a structured process comprising a literature review, cross-cultural adaptation, expert Delphi consultations, and pilot survey. Subsequently, a community hospital-based case-control study was executed to evaluate the validity and reliability of the instrument. Conducted between July and November 2024, the validation study enrolled 196 children aged 0–6 years along with their guardians at the General Pediatrics and Pediatric Allergy Clinic of Gumei Community Health Service Center in Minhang District, Shanghai.

#### 2.2.1 Questionnaire development and localization

The questionnaire was adapted from three validated questionnaires: ISAAC, CECAQ and YCAR-Q. Specifically: FA items were primarily derived from CECAQ and the EuroPrevall project (10). Asthma and AD sections were adapted from ISAAC and CECAQ. AR components were modified from ISAAC protocols and YCAR-Q.

All items underwent Brislin translation model (11) for cross-cultural adaptation, comprising three steps:

1. Translation: two independent bilingual translators (Master of Public Health candidates at Fudan University with CET-6 certification) translated the source items into Chinese.

2. Integration: discrepancies were resolved iteratively to form a unified Chinese draft.

3. Back-translation: A bilingual expert in preventive medicine and health education back-translated the Chinese version into English to verify semantic and conceptual equivalence, followed by expert panel discussions involving pediatricians and allergists to finalize the preliminary scale. The preliminary scale underwent two rounds of expert consultation with six specialists in diverse research fields and scale development. Six experts across disciplines (including areas such as maternal and child health, pediatric asthma, allergic rhinitis, chronic cough in children, dermatology, pediatric nutrition, and neonatology) participated in two rounds of expert consultations to evaluate the linguistic accuracy of localized items and their diagnostic necessity for targeted allergic diseases. Objectives included evaluating: linguistic equivalence of translated items, relevance of items for diagnosing allergic diseases in 0–6–year–olds, and omission of critical symptoms.

Subsequently, a pilot survey was conducted among the guardians of 28 children aged 0 to 6 years to evaluate the clarity, comprehensibility, and completion time of the questionnaire. Final revisions were made based on the feedback received.

#### 2.2.2 Participants

Sample size calculation was based on sensitivity and specificity (both set at 0.85), with α = 0.05 and β = 0.8, yielding a required total of 196 participants (98 cases and 98 controls). Considering the critical phases of early immunological transition, the control group was assembled using a 1:1 frequency matching strategy based on sex and specific age strata (0–1 year, 1–3 years, and 3–6 years). Power calculation was based on across all four conditions and through the formula (12).

*TP* + *FN* = *Z*<*uscore*>(α/2)ˆ2 (*SN*(1 − *SN*))/*Wˆ*2

*FP* + *TN* = *Z*<*uscore*>(ω/2)ˆ2 (*SP*(1 − *SP*))/(*Wˆ*2)

#### 2.2.3 Validwation and gold standards

*Inclusion criteria:* children aged 0–6 years, guardians with sufficient language proficiency to complete the questionnaire independently.

*Exclusion criteria:* children with complex comorbidities.

To minimize response and expectation biases, a double-blind design was strictly implemented in this study. The participating children underwent physician diagnostic evaluation only after their guardians completed the questionnaire, and the questionnaire results were withheld from both the diagnosing physicians and the guardians throughout the diagnostic process. The physician diagnostic evaluation occurred on the same day, immediately following the completion of the questionnaire (time interval of 0–1 h). Three physicians participated, all trained uniformly using a quality control manual to standardize understanding of study objectives and implementation protocols. Diagnoses adhered to predefined gold-standard criteria. To ensure diagnostic accuracy, all participants' test results and electronic medical records (EMRs) were retained. Diagnoses were based on gold-standard criteria for the four allergic diseases:

IgE-mediated FA: according to the EAACI (European Academy of Allergy and Clinical Immunology) guidelines, requiring a convincing clinical history of reproducible symptoms upon ingestion of the suspected food, corroborated by objective evidence of sensitization which was defined as a positive Skin Prick Test (SPT) (wheal diameter ≥ 3 mm larger than the negative control) using allergen extracts or fresh foods, or specific IgE (sIgE) levels ≥ 0.35 kU/L. Oral food challenges (OFC) were performed solely in equivocal cases to definitively confirm the diagnosis (13, 14).

Asthma: according to the Guidelines for bronchial asthma prevent and management (2020 edition) Asthma group of Chinese Thoracic Society (15). Symptoms and signs: Recurrent episodes of wheezing, shortness of breath, chest tightness, and coughing; Objective evidence of variable airflow limitation (any one of the following criteria): Positive bronchodilator test, Positive bronchial provocation test, Peak expiratory flow (PEF) variability >10% (excluding respiratory infections). For children under 3 years of age, or those unable to perform spirometry, the diagnosis relied on a compatible clinical history, documentation of recurrent wheezing episodes, and a definitive clinical response to a therapeutic trial of short-acting bronchodilators and inhaled corticosteroids in accordance with Global Initiative for Asthma (GINA) guidelines (16).

Atopic dermatitis: UK working party criteria for AD: including history of flexural involvement, history of a dry skin, personal history of asthma, history of a pruritic skin condition, and visible flexural dermatitis (17).

Allergic rhinitis: According to the ICRA2023 (International Consensus Statement on Allergy and Rhinology), Skin prick test (SPT) /Serum total and specific IgE (18, 19).

Control group: subjects who could not be diagnosed with the aforementioned allergic diseases based on the same gold standard were enrolled in the control group. Furthermore, children with a history of other allergic conditions (e.g., drug allergies, urticaria) were strictly excluded to ensure the immunological purity of the control arm.

**Figure 2:**
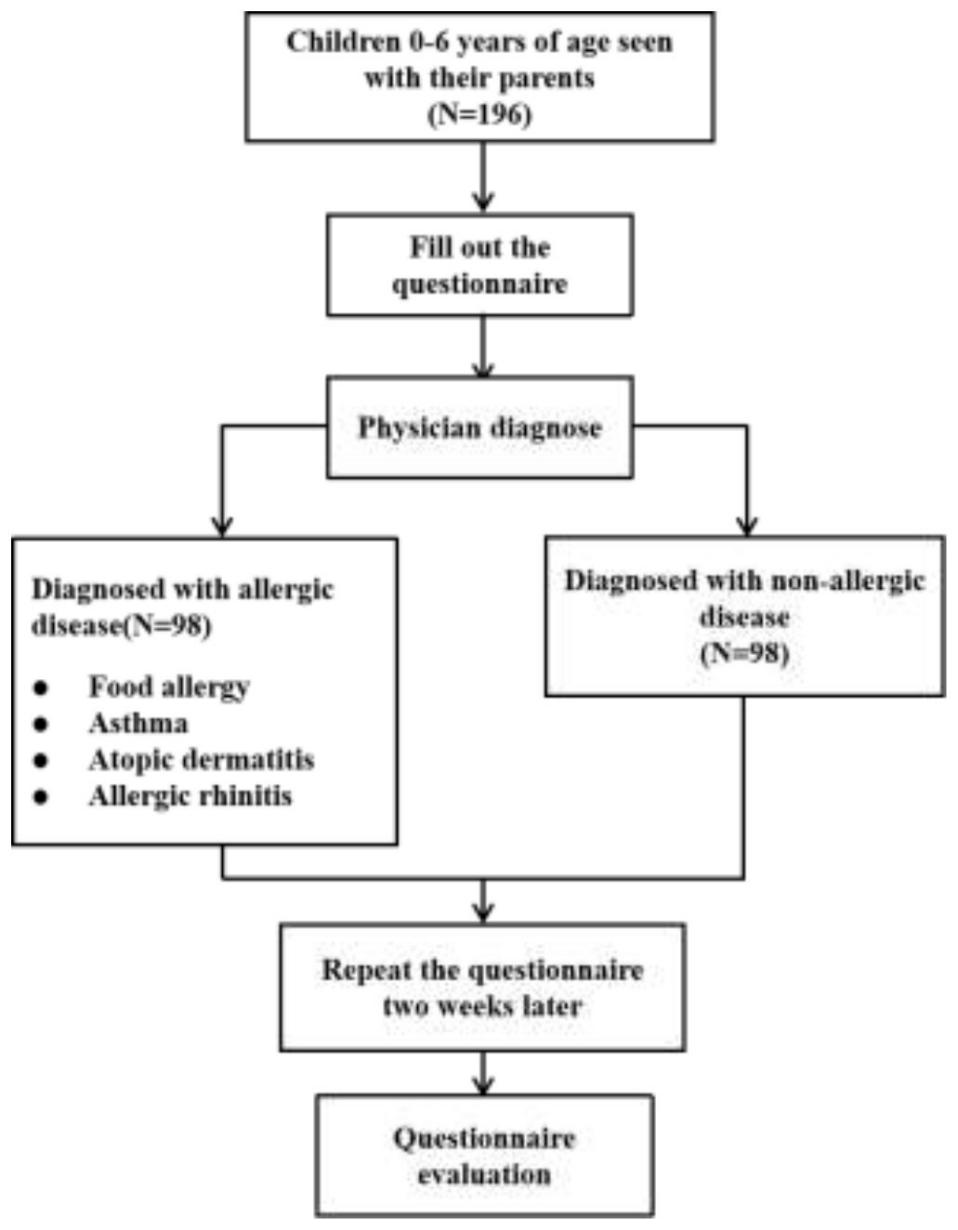
Recruitment process.

A follow-up was conducted among the 196 participants who completed the initial screening to evaluate the consistency of results, with 172 questionnaires successfully retrieved, yielding a follow-up rate of 88% (Figure 2). To evaluate potential attrition bias, we compared the baseline characteristics of the initial screening cohort (*N* = 196) with those of the successfully followed-up cohort (*N* = 172). No statistically significant differences were observed between the two cohorts regarding age (*P* = 0.773), gender distribution (*P* = 0.881), or clinical allergy profiles (*P* = 0.980), Table S1. These results indicate that the follow-up sample remained representative of the baseline population, suggesting that attrition bias was negligible and did not compromise the validity of the reliability estimates.

### 2.3 Phase II: cross-sectional survey

A district-wide cross-sectional survey was conducted in Minhang District, Shanghai from June to October 2025, to apply the validated CACSQ. Based on the geographic distribution characteristics of the pediatric population in Minhang District, a multi-stage stratified random cluster sampling method was employed. First, the district was partitioned into three geographic strata—Southern, Central, and Northern—from which eight community health service centers and 15 kindergartens/childcare institutions were randomly selected. Subsequently, all children aged 0–6 years within the designated facilities were enrolled via cluster sampling. Concurrently, to account for variations in institutional quality rankings, the sampled facilities were structurally selected to encompass demonstration, tier-1, and tier-2 kindergartens.

#### 2.3.1 Participants

The sample size was estimated based on an assumed allergic disease prevalence of 20% (20), a margin of error of 2%, and a confidence level of 95%, requiring a minimum of 2,000 participants. Our final sample exceeded this requirement.

Before the epidemiological investigation, we held online and offline meetings with administrators of selected institutions and physicians from community health centers to detail the study plan (including objectives, methodology, implementation, and feedback of results). Subsequently, school administrators notified parents to ensure full understanding and cooperation, while community health centers promoted participation through posters and physician education. Nearly all sampled parents completed the questionnaire, with 3,133 initial responses. After excluding children over 6 years old or without guardian consent, 3,064 (97.8%) children aged 0–6 were ultimately included.

#### 2.3.2 Questionnaire and variables

Questions regarding the general sociodemographic characteristics of the children and their parents, socioeconomic status (SES, evaluated via parental educational attainment and household housing status), personal and familial histories of allergy and atopic diseases, prenatal and gestational exposures, children's lifestyle habits, early-life home environmental exposures, as well as early-life infections and history of medication use were similar to those used for the Swedish Dampness in Buildings and Health (DBH) (21) study and the China, Children, Homes, Health (CCHH) study (22). All adapted items were translated into Mandarin. All items were translated into Mandarin with appropriate cultural contents.

#### 2.3.3 Assessment of allergic diseases

The validated CACSQ was developed to assess for four types of allergic diseases in children aged 0–6 years. Disease classification was based on the pre-established CACSQ cutoff criteria derived from Phase I validation.

### 2.4 Statistical analysis

Categorical variables were described using frequencies and percentages, and differences in the prevalence of allergic diseases across different populations were assessed using the Pearson chi-square test. For continuous variables, those following a normal distribution were described as mean ± standard deviation (SD), differences between diseased and non-diseased populations were evaluated using Welch's *t*-test or *t*'-test.

**Scale performance (phase 1):** receiver operating characteristic (ROC) curves and the area under the curve (AUC) were used to determine the optimal cut-off values based on Youden index maximization. Accuracy metrics included sensitivity, specificity, and positive/negative likelihood ratios (LR+/LR-). Internal consistency was assessed using Cronbach's α and split-half reliability (with Spearman-Brown correction). Test-retest reliability was evaluated using Cohen's Kappa coefficients.

**Association analyses (phase 2):** Univariable logistic regression models were used to calculate the odds ratios (OR) and 95% confidence intervals (CI) for factors such as child sex, age, birth weight, mode of delivery, breastfeeding duration, only child status, and family history of allergies. Multivariable logistic regression models were employed to calculate the adjusted odds ratios (AOR) and 95% CIs after controlling for confounding factors.

*Subgroup and sensitivity analyses:* Subgroup analyses were conducted based on a priori considerations of potential effect modification, as informed by tests of statistical interaction. Given the distinct peak age ranges and immune maturation patterns within the 0–6–year spectrum, participants were stratified into two age groups: the window period (< 3 years) and the transitional period (3–6 years). Additional stratification was performed by sex. For sensitivity analysis, we assessed the robustness of our findings by adjusting for the number of children in the household and by applying the standard deviation method to account for extreme values in birth weight.

All statistical analyses were performed using R version 4.5.2 (R Core Team, 2026; R Foundation for Statistical Computing, Vienna, Austria. URL https://www.R-project.org/).

## 3 Results

### 3.1 Phase 1: psychometric performance of the CACSQ

#### 3.1.1 Scale development and revision

##### 3.1.1.1 Expert consultation

In the first round, items were evaluated for relevance and clarity. An item was automatically retained if it achieved >80% consensus agreement among the panel of experts. For items with divergent opinions, research team discussions were conducted, and revisions were made based on expert feedback. A total of 17 feedback comments were collected, and seven actionable suggestions were incorporated into the preliminary scale after panel discussion. All items were approved in the second round of expert review. The translation quality of the questionnaire was not questioned.

During the development of the preliminary scale, dermatologists and pediatric practitioners were consulted to remove items that could not serve as valid diagnostic criteria for children aged 0–6 years. Regarding the FA section, Item three was adapted with reference to the EuroPrevall scale, expanding the range of pediatric food allergy symptoms to include ‘dizziness, headache, syncope, and joint stiffness'. Item four incorporated ‘fish/shrimp, nuts, and dairy products' — allergens more prevalent in the Chinese population (23).

The asthma section was refined by removing redundant content, specifically inquiries about past symptoms, and now only assesses symptoms reported by guardians within the last 12 months. The classification of wheezing episodes was further stratified into: ‘ < 1, 1–3, 4–12, and >12 times'.

In the AD section, the term ‘itching' was replaced with the more clinically precise ‘pruritus'. The criterion ‘pruritus lasting at least 6 months' was excluded as it is not essential for pediatric AD diagnosis.

For the AR section, based on clinical expert consensus, ‘mouth breathing' was added to the symptom inquiries in Item 20, while ‘family allergy' was excluded as a non-essential diagnostic criterion for AR.

##### 3.1.1.2 Pilot survey

Twenty-eight participants were recruited for pilot testing. All respondents deemed the questionnaire clear and comprehensible. Based on feedback, the suggested time range for the frequency of rash occurrence added to item 14, the frequency of symptoms ‘in the last 12 months' was asked. The average completion time was 206 s (range: 74–893 s).

#### 3.1.2 Validation

The scale comprises four disease-specific subscales encompassing a total of 24 items. Specifically, items 1–7 evaluate FA status, items 8–12 assess asthma status, items 13–19 evaluate AD status, and items 20–24 assess AR status.

Total scores for each subscale are computed by aggregating individual item scores based on a standardized assignment protocol: dichotomous items are scored 1 for “yes” and 0 for “no”; ordinal items are graded 0, 1, 2, or 3 points in ascending order of clinical severity based on a priori clinical consensus regarding symptom severity and frequency (from mild to severe), as determined through the two-round expert consultation process; and multiple-choice items are scored based on symptom frequency, yielding 0 points (asymptomatic/unknown), 1 point (one symptom), 2 points (two to three symptoms), or 3 points (more than three symptoms).

##### 3.1.2.1 Characteristics of participants

The validation sample (*n* = 196) comprised 98 cases (mean age 3.24 ± 1.63 years; 67.3% male) and 98 controls (mean age 2.97 ± 1.69 years; 57.1% male), with no statistically significant differences in age (*P* = 0.245) or sex (*P* = 0.092) between the groups (Table 1). Within the case group, the distribution of gold-standard clinical diagnoses was: FA: 27 (13.8%), asthma: 28 (14.3%), AD: 52 (26.5%), and AR: 74 (37.8%). Comparatively, the corresponding disease distribution identified by the CACSQ definition was: FA: 42 (21.4%), asthma: 29 (14.8%), AD: 70 (35.7%), and AR: 62 (31.6%), Table 2. Co-occurrence was common: 20.9% had two concurrent allergic conditions, 7.7% had three, and 2.0% had all four.

**Table 1:** Characteristics of participants.

Variable	Children with diagnosed allergies (*n* = 98)	Children without diagnosed allergies (*n* = 98)	*P*-value
**Age, Years**			
Mean (S ± D)	3.24 (±1.63)	2.97 (±1.69)	0.245
**Gender, *n* (%)**			
Male	66 (33.7)	56 (21.4)	
Female	32 (16.3)	42 (28.6)	0.092

Non-normally distributed data were analyzed using non-parametric tests, while chi-square tests were applied to 2 × 2 contingency tables.

**Table 2:** Allergic conditions identified by the questionnaire and physician diagnosis.

Allergic conditions	Defined by questionnaire *n* (%)	Diagnosed by physician *n* (%)
Food allergy	42 (21.4)	27 (13.8)
Asthma	29 (14.8)	28 (14.3)
Allergic dermatitis	70 (35.7)	52 (26.5)
Allergic rhinitis	62 (31.6)	74 (37.8)
Any allergy	116 (59.2)	98 (50.0)

##### 3.1.2.2 Scale evaluation

**Figure 3:**
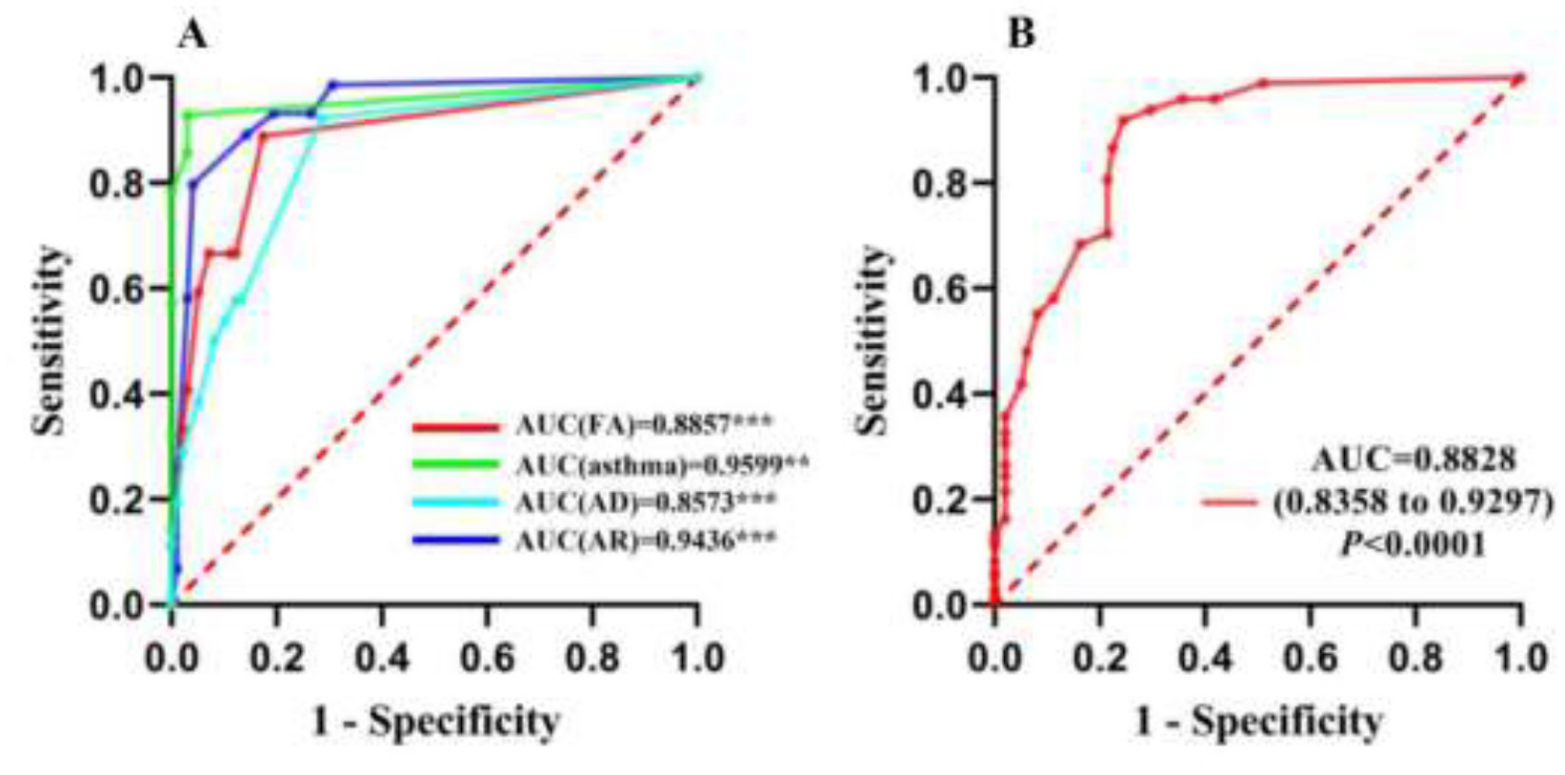
**(A)** ROC analysis of the FA (food allergy), asthma, AD (atopic dermatitis), and AR (allergic rhinitis) subscales. **(B)** ROC analysis of CACSQ. ^**^ indicates *P* 0.01, ^***^ indicates *P* < 0.001.

Optimal diagnostic cut-offs and evaluation metrics for CACSQ and each disease subscale are summarized in Tables 3 and 4. Psychometric evaluation verified that the CACSQ possesses validity and reliability across all target conditions (all *P* < 0.001), ROC analysis demonstrated strong discriminative performance, with the AUC ranging from 0.8573 to 0.9599 (Figure 3). Specifically, the optimal thresholds and corresponding diagnostic parameters were identified as follows: FA (cutoff: 2 points; total score: 12; AUC = 0.8857; sensitivity/specificity: 0.89/0.83); asthma (cutoff: 1 point; total score: 9; AUC = 0.9599; sensitivity/specificity: 0.93/0.97); AD (cutoff: 2 points; total score: 15; AUC = 0.8573; sensitivity/specificity: 0.92/0.71); and AR (cutoff: 5 points; total score: 9; AUC = 0.9436; sensitivity/specificity: 0.80/0.96).

**Table 3:** Authenticity evaluation of CACSQ.

Disease condition	Cut-off Points	Sensitivity (95% CI)	Specificity (95% CI)	LR+	LR-
Food allergy	≥2	0.89 (0.72–0.96)	0.83 (0.74–0.89)	5.12	0.19
Asthma	≥1	0.93 (0.77–0.99)	0.97 (0.91–0.99)	30.34	0.03
Atopic dermatitis	≥2	0.92 (0.72–0.96)	0.71 (0.63–0.79)	3.23	0.11
Allergic rhinitis	≥5	0.80 (0.69–0.87)	0.96 (0.90–0.98)	19.54	0.21
Combined	≥6	0.92 (0.85–0.96)	0.76 (0.66–0.83)	3.75	0.67

**Table 4:** Consistency evaluation of CACSQ.

Disease condition	*N* of Items	Cronbach's alpha	Spearman-brown Coefficient	Kappa coefficient
Food allergy	6	0.882	0.910	0.821
Asthma	6	0.853	0.828	0.884
Atopic dermatitis	7	0.889	0.828	0.752
Allergic rhinitis	5	0.845	0.887	0.684
Questionnaire	24	0.869	0.660	0.698

Reliability evaluations showed excellent internal consistency and reproducibility across all subscales. Cronbach's α ranged from 0.845 to 0.889, split-half reliability coefficients ranged from 0.828 to 0.910, and test-retest Kappa coefficients indicated substantial to almost perfect agreement (0.684 to 0.884), as shown in Table 2.

### 3.2 Phase 2: cross-sectional survey results

#### 3.2.1 Characteristics of the sample

Table 5 depicts the summary statistics of characteristics among 3,064 children, and overall 3,064 children aged 0–6 with an average age of 3.2 ± 2.1 years, including 1,626 boys (*53.1%*) and 1,438 girls (*46.9%*). Based on the validated CACSQ screening, the overall questionnaire-identified prevalence rates of FA, asthma, AD, and AR among children were 14.7%, 8.4%, 18.9%, and 20.0%, respectively. Notably, the incidence of asthma was significantly higher in boys (*P* < 0.001).

**Table 5:** Summary statistics of characteristics and the prevalence of asthma and allergic diseases among different groups.

Variable	Total, *n* (%)	Food allergy *n* (%)	*P* Value	Asthma *n* (%)	*P* Value	Atopic dermatitis n (%)	*P* Value	Allergic rhinitis *n* (%)	*P* Value
	3064	451 (14.7%)		256 (8.4%)		578 (18.9%)		612 (20.0%)	
Gender			0.586		< 0.001		0.946		0.573
Girls	1438 (46.9%)	217 (15.1%)		86 (6.0%)		272 (18.9%)		281 (19.5%)	
Boys	1626 (53.1%)	234 (14.4%)		170 (10.5%)		306 (18.8%)		331 (20.4%)	
Age (year)			< 0.001		< 0.001		< 0.001		< 0.001
≤ 1	742 (24.2%)	42 (5.7%)		17 (2.3%)		153 (20.6%)		27 (3.6%)	
1–3	265 (8.6%)	51 (19.2%)		7 (2.6%)		69 (26.0%)		20 (7.5%)	
> 3	250 (8.2%)	36 (14.4%)		22 (8.8%)		52 (20.8%)		55 (22.0%)	
Race			0.027		0.386		0.884		0.099
Han Ethnicity	2961 (96.6%)	428 (14.5%)		245 (8.3%)		558 (18.8%)		598 (20.2%)	
Others	103 (3.4%)	23 (22.3%)		11 (10.7%)		20 (19.4%)		14 (13.6%)	
Number of children			0.138		0.844		0.010		0.052
0	1904 (62.1%)	302 (15.9%)		154 (8.1%)		391 (20.5%)		408 (21.4%)	
1	1040 (33.9%)	134 (12.9%)		90 (8.7%)		164 (15.8%)		182 (17.5%)	
2	107 (3.5%)	14 (13.1%)		11 (10.3%)		22 (20.6%)		21 (19.6%)	
≥3	13 (0.4%)	1 (7.7%)		1 (7.7%)		1 (7.7%)		1 (7.7%)	
Degree of mother			< 0.001		0.057		0.222		< 0.001
Junior high school and below	136 (4.4%)	6 (4.4%)		4 (2.9%)		25 (18.4%)		10 (7.4%)	
High/vocational high school	250 (8.2%)	14 (5.6%)		16 (6.4%)		36 (14.4%)		26 (10.4%)	
Undergraduate	2059 (67.2%)	323 (15.7%)		178 (8.6%)		390 (18.9%)		445 (21.6%)	
Postgraduate	619 (20.2%)	108 (17.4%)		58 (9.4%)		127 (20.5%)		131 (21.2%)	
Degree of father			< 0.001		0.107		0.018		< 0.001
Junior high school and below	130 (4.2%)	11 (8.5%)		6 (4.6%)		18 (13.8%)		9 (6.9%)	
Undergraduate	1985 (64.8%)	303 (15.3%)		179 (9.0%)		387 (19.5%)		426 (21.5%)	
Postgraduate	683 (22.3%)	117 (17.1%)		56 (8.2%)		139 (20.4%)		152 (22.3%)	
High/vocational high school	266 (8.7%)	20 (7.5%)		15 (5.6%)		34 (12.8%)		25 (9.4%)	
Premature birth			0.005		0.312		0.746		< 0.001
No	333 (10.9%)	32 (9.6%)		23 (6.9%)		65 (19.5%)		39 (11.7%)	
Yes	2731 (89.1%)	419 (15.3%)		233 (8.5%)		513 (18.8%)		573 (21.0%)	
Birth mode			0.156		0.749		0.031		0.445
Vaginal delivery	1419 (46.3%)	195 (13.7%)		121 (8.5%)		291 (20.5%)		275 (19.4%)	
Cesarean section	1645 (53.7%)	256 (15.6%)		135 (8.2%)		287 (17.4%)		337 (20.5%)	
Family allergy history			< 0.001		< 0.001		< 0.001		< 0.001
No	1482 (48.4%)	122 (8.2%)		66 (4.5%)		179 (12.1%)		156 (10.5%)	
Yes	1582 (51.6%)	329 (20.8%)		190 (12.0%)		399 (25.2%)		456 (28.8%)	
Daily sleep time of children (hours)			< 0.001		< 0.001		0.003		< 0.001
< 10 h	1552 (50.7%)	261 (16.8%)		161 (10.4%)		256 (16.5%)		387 (24.9%)	
10–12 h	860 (28.1%)	140 (16.3%)		78 (9.1%)		188 (21.9%)		197 (22.9%)	
≥12 h	652 (21.3%)	50 (7.7%)		17 (2.6%)		134 (20.6%)		28 (4.3%)	
Residential floor			0.186		0.716		0.049		0.016
1–3 floors	1104 (36.0%)	147 (13.3%)		95 (8.6%)		183 (16.6%)		207 (18.8%)	
4–6 floors	1066 (34.8%)	159 (14.9%)		92 (8.6%)		218 (20.5%)		243 (22.8%)	
≥7 floors	894 (29.2%)	145 (16.2%)		69 (7.7%)		177 (19.8%)		162 (18.1%)	
Decoration situation of the permanent residence			0.001		< 0.001		0.575		< 0.001
No	2385 (77.8%)	320 (13.4%)		177 (7.4%)		448 (18.8%)		442 (18.5%)	
Before pregnancy	184 (6.0%)	38 (20.7%)		15 (8.2%)		29 (15.8%)		40 (21.7%)	
during pregnancy	82 (2.7%)	18 (22.0%)		9 (11.0%)		16 (19.5%)		10 (12.2%)	
after birth	413 (13.5%)	75 (18.2%)		55 (13.3%)		85 (20.6%)		120 (29.1%)	
Wall materials for children's rooms			0.219		0.145		0.014		0.002
Seaweed mud	25 (0.8%)	4 (16.0%)		3 (12.0%)		6 (24.0%)		7 (28.0%)	
Wallpaper	396 (12.9%)	57 (14.4%)		24 (6.1%)		74 (18.7%)		80 (20.2%)	
water-based paint/latex paint	1427 (46.6%)	232 (16.3%)		139 (9.7%)		270 (18.9%)		322 (22.6%)	
oil-based paint	36 (1.2%)	5 (13.9%)		2 (5.6%)		10 (27.8%)		4 (11.1%)	
wooden board	47 (1.5%)	3 (6.4%)		5 (10.6%)		5 (10.6%)		8 (17.0%)	
lime/cement	91 (3.0%)	10 (11.0%)		9 (9.9%)		9 (9.9%)		14 (15.4%)	
wall painting	916 (29.9%)	128 (14.0%)		68 (7.4%)		191 (20.9%)		166 (18.1%)	
Others	126 (4.1%)	12 (9.5%)		6 (4.8%)		13 (10.3%)		11 (8.7%)	
Mother's exposure to secondhand smoke from family members during pregnancy			0.015		0.464		0.187		0.366
No	2609 (85.2%)	367 (14.1%)		214 (8.2%)		482 (18.5%)		514 (19.7%)	
Yes	455 (14.8%)	84 (18.5%)		42 (9.2%)		96 (21.1%)		98 (21.5%)	
Mother smoking			0.984		0.006		0.263		0.941
No	3010 (98.2%)	443 (14.7%)		246 (8.2%)		571 (19.0%)		601 (20.0%)	
Yes	54 (1.8%)	8 (14.8%)		10 (18.5%)		7 (13.0%)		11 (20.4%)	

Data were presented with frequency and percentage; *p*-value was calculated by Pearson's chi-squared (χ2) test.

**Figure 4:**
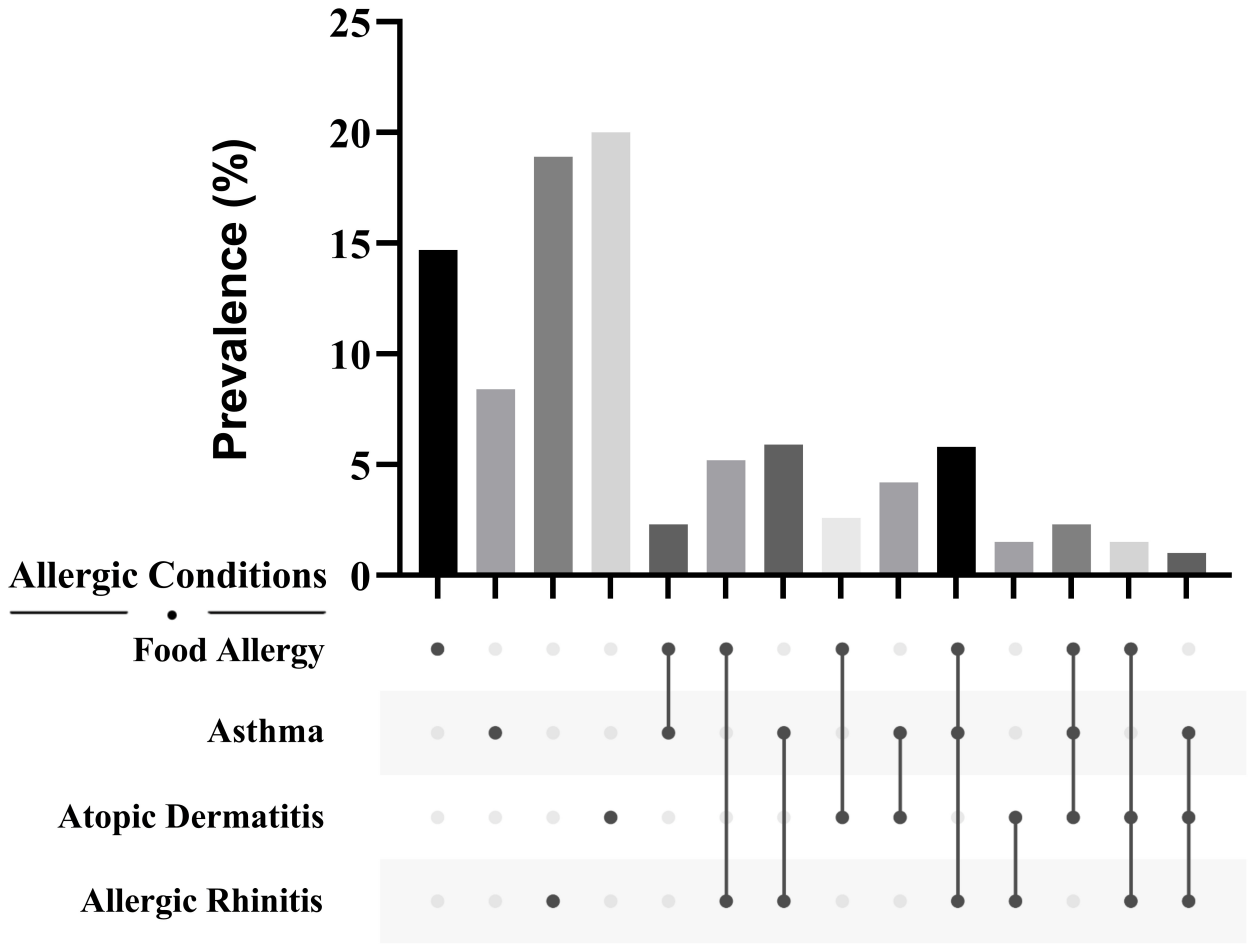
The comorbidity of allergic diseases in children. The incidence rate on the vertical coordinate is an exact set of each classification. AR, allergic rhinitis; AD, atopic dermatitis; FA, food allergies.

Analysis revealed frequent co-occurrence among FA, AD, and AR, a significant increase in prevalence was observed in children aged 3–6 years (*P* < 0.001). Specifically, the prevalence rates of children suffering from FA and AD simultaneously, FA and AR, and AD and AR were 5.2%, 5.9%, and 5.8% respectively. (Figure 4 and Supplementary Table S1).

### 3.3 Influencing factors of allergies in children

Table 6 presents the results of logistic regression models about correlative factors for childhood asthma and allergic diseases. Overall, a family history of allergies, longer annual duration of air conditioner use, a history of antibiotic treatment, and a history of pneumonia were associated with higher OR of asthma and allergic diseases. For instance, the AORs for children had a family history of allergies were 2.29 (*95%CI: 1.81–2.90*) for FA, 2.17 (*95%CI: 1.58–2.98*) for asthma, 2.32 (*95%CI: 1.88–2.85*) for AD, and 2.83 (*95%CI: 2.27–3.51*) for AR. In contrast, having siblings (particularly one sibling) and an average sleep duration of >12 h per day were associated with lower OR. The AORs for children have an average sleep duration of >12 h were FA (*AOR* = *0.58, 95%CI: 0.38–0.88*), asthma (*AOR* = *0.46, 95%CI: 0.25–0.88*), and AR (*AOR* = *0.38, 95%CI: 0.23–0.60*). Similarly, the AORs for those with one sibling were FA (*AOR* = *0.79, 95%CI: 0.62–0.99*), AD (*AOR* = *0.81, 95%CI: 0.65–0.99*), and AR (*AOR* = *0.67, 95%CI: 0.54–0.84*).

**Table 6:** Results of univariate and multivariate logistics regression analyses between childhood allergy and independent variables.

Variable	Food allergy		Asthma		Atopic dermatitis		Allergic rhinitis	
	crude OR	adjusted OR	crude OR	adjusted OR	crude OR	adjusted OR	crude OR	adjusted OR
Age/years	**1.14 (1.09**–**1.20)**	1.02 (0.94–1.10)	**1.24 (1.16**–**1.33)**	1.02 (0.92–1.14)	**0.91 (0.87**–**0.95)**	**0.85 (0.80**–**0.92)**	**1.38 (1.31**–**1.45)**	**1.23 (1.14**–**1.32)**
Air conditioners usage/months	**1.12 (1.07**–**1.17)**	**1.07 (1.02**–**1.13)**	**1.14 (1.07**–**1.21)**	**1.09 (1.02**–**1.18)**	**1.10 (1.06**–**1.15)**	**1.06 (1.01**–**1.11)**	**1.11 (1.06**–**1.16)**	**1.07 (1.02**–**1.13)**
Gender (girls)								
Boys	0.95 (0.77–1.16)	0.95 (0.76–1.17)	**1.84 (1.40**–**2.40)**	**1.97 (1.47**–**2.64)**	0.99 (0.83–1.19)	0.99 (0.82–1.20)	1.05 (0.88–1.26)	1.06 (0.87–1.29)
Race (Han Ethnicity)								
Ethnic minority	**1.70 (1.06**–**2.74)**	**1.85 (1.12**–**3.04)**	**1.81 (1.09**–**3.01)**	1.33 (0.70–2.51)	1.31 (0.68–2.54)	1.29 (0.65–2.55)	1.04 (0.63–1.71)	1.10 (0.66–1.82)
**Number of children** **(****1****)**								
2	**0.78 (0.63**–**0.98)**	**0.79 (0.62**–**0.99)**	1.08 (0.82–1.41)	1.09 (0.80–1.47)	**0.72 (0.59-0.89)**	**0.81 (0.65**–**0.99)**	**0.78 (0.64**–**0.94)**	**0.67 (0.54**–**0.84)**
3	0.80 (0.45–1.42)	1.11 (0.60–2.05)	1.30 (0.68–2.48)	1.39 (0.65–2.94)	1.00 (0.62–1.62)	1.08 (0.65–1.82)	0.90 (0.55–1.46)	1.25 (0.71–2.20)
≥4	0.44 (0.06–3.41)	0.82 (0.09–7.19)	0.95 (0.12–7.33)	1.20 (0.12–11.80)	0.32 (0.04–2.49)	0.37 (0.05–3.04)	0.31 (0.04–2.36)	0.39 (0.04–3.95)
**Degree of mother (Junior high school and below)**								
High/vocational high school	1.29 (0.48–3.42)	1.18 (0.41–3.41)	2.26 (0.74–6.89)	1.62 (0.46–5.71)	0.75 (0.43–1.31)	0.59 (0.31–1.12)	1.46 (0.68–3.13)	0.84 (0.35–1.97)
Undergraduate	**4.03 (1.76**–**9.22)**	**2.99 (1.12**–**7.99)**	**3.12 (1.14**–**8.54)**	1.75 (0.52–5.91)	1.04 (0.66–1.62)	0.59 (0.33–1.06)	**3.47 (1.81**–**6.67)**	1.16 (0.52–2.59)
Postgraduate	**4.58 (1.97-10.65)**	**3.26 (1.18**–**9.06)**	**3.41 (1.22**–**9.56)**	2.18 (0.61–7.81)	1.15 (0.71–1.84)	0.62 (0.32–1.17)	**3.38 (1.73**–**6.62)**	1.12 (0.48–2.60)
**Degree of Father (Junior high school and below)**								
High/vocational high school	0.88 (0.41–1.90)	0.62 (0.26–1.48)	1.24 (0.47–3.26)	0.88 (0.29–2.70)	0.91 (0.49–1.69)	1.30 (0.65–2.61)	1.39 (0.63–3.08)	1.45 (0.59–3.56)
Undergraduate	**1.95 (1.04**–**3.66)**	0.71 (0.32–1.57)	2.05 (0.89–4.71)	0.97 (0.34–2.78)	1.51 (0.90–2.51)	1.83 (0.95–3.53)	**3.67 (1.85**–**7.29)**	**2.35 (1.01**–**5.48)**
Postgraduate	**2.24 (1.17**–**4.28)**	0.74 (0.32–1.71)	1.85 (0.78–4.38)	0.69 (0.23–2.11)	1.59 (0.93–2.70)	1.83 (0.91–3.71)	**3.85 (1.91**–**7.76)**	**2.55 (1.05**–**6.18)**
**Premature birth (No)**								
Yes	**1.70 (1.17**–**2.49)**	1.48 (0.99–2.22)	1.26 (0.81–1.96)	1.15 (0.70–1.90)	0.95 (0.72–1.27)	1.04 (0.76–1.42)	**2.00 (1.42**–**2.83)**	**1.61 (1.09**–**2.37)**
**Birth mode (Vaginal delivery)**								
Cesarean section	1.16 (0.95–1.42)	1.09 (0.88–1.35)	0.96 (0.74–1.24)	0.89 (0.67–1.18)	**0.82 (0.68**–**0.98)**	0.85 (0.70–1.03)	1.07 (0.90–1.28)	0.94 (0.77–1.15)
**Has the child ever had pneumonia (No)**								
Yes	**2.27 (1.84**–**2.80)**	**1.63 (1.28**–**2.06)**	**5.77 (4.42**–**7.54)**	**3.66 (2.72**–**4.95)**	1.21 (0.99–1.49)	1.08 (0.86–1.37)	**2.68 (2.22**–**3.23)**	**1.55 (1.25-1.92)**
**Has the child ever had Pediatric inflammation (No)**								
Yes	**1.60 (1.11**–**2.30)**	1.21 (0.82–1.79)	**1.91 (1.25**–**2.94)**	1.18 (0.74–1.91)	**1.54 (1.10**–**2.16)**	**1.65 (1.15**–**2.38)**	**2.45 (1.80**–**3.34)**	1.32 (0.94-1.87)
**Has the child ever received antibiotic treatment (No)**								
Yes	**2.33 (1.86**–**2.92)**	**1.38 (1.04**–**1.83)**	**5.99 (4.06**–**8.83)**	**2.59 (1.64**–**4.10)**	1.21 (1.00–1.46)	**1.32 (1.03**–**1.69)**	**3.87 (3.12**–**4.82)**	**1.69 (1.30-2.19)**
**Family history of allergy (No)**								
Yes	**2.93 (2.35**–**3.65)**	**2.30 (1.82**–**2.90)**	**2.93 (2.19**–**3.91)**	**2.15 (1.57**–**2.95)**	**2.46 (2.02**–**2.98)**	**2.35 (1.91**–**2.89)**	**3.44 (2.82**–**4.20)**	**2.84 (2.28-3.52)**
**Fresh air systems usage frequency (No)**								
Sometimes	0.88 (0.63–1.23)	0.86 (0.60–1.25)	1.04 (0.69–1.56)	1.22 (0.77–1.93)	0.75 (0.55–1.03)	0.86 (0.61–1.20)	**0.69 (0.51**–**0.95)**	**0.69 (0.48-0.98)**
Often	1.02 (0.67–1.55)	1.19 (0.75–1.89)	**0.35 (0.16**–**0.81)**	**0.34 (0.14**–**0.82)**	0.70 (0.46–1.07)	0.66 (0.42–1.05)	0.68 (0.45–1.02)	0.76 (0.48-1.23)
**Incense/air fresheners usage frequency (No)**								
Sometimes	1.09 (0.83–1.44)	0.90 (0.67–1.23)	1.16 (0.82–1.65)	0.98 (0.66–1.45)	0.86 (0.66–1.12)	0.93 (0.70–1.25)	1.40 (1.10–1.77)	1.12 (0.85-1.47)
Often	0.81 (0.45–1.45)	0.97 (0.51–1.84)	0.78 (0.36–1.70)	0.74 (0.31–1.77)	0.68 (0.39–1.18)	0.85 (0.47–1.54)	1.52 (0.98–2.37)	**1.98 (1.17-3.36)**
**Open the windows for ventilation within one day (0 times)**								
1	0.64 (0.21–1.99)	0.45 (0.14–1.49)	0.44 (0.14–1.37)	**0.17 (0.05**–**0.61)**	0.40 (0.15–1.06)	**0.34 (0.12**–**0.95)**	0.77 (0.25–2.38)	0.31 (0.09–1.11)
2	0.63 (0.20–1.95)	0.56 (0.17–1.85)	**0.31 (0.10**–**0.98)**	**0.15 (0.04**–**0.54)**	**0.37 (0.14**–**0.99)**	**0.33 (0.12**–**0.93)**	0.75 (0.24–2.32)	0.40 (0.11–1.39)
≥3	0.58 (0.19–1.78)	0.47 (0.14–1.53)	0.28 (0.09–0.88)	0.10 (0.03–0.37)	0.35 (0.13–0.90)	0.34 (0.12–0.95)	0.95 (0.31–2.91)	0.39 (0.11–1.37)
**Daily exercise time for children (**<**2 h)**					
≥2h	1.09 (0.89–1.34)	0.97 (0.78–1.20)	0.94 (0.72–1.22)	0.80 (0.60–1.07)	0.94 (0.78–1.13)	0.98 (0.80–1.19)	0.95 (0.79–1.14)	**0.78 (0.64**–**0.95)**
**Daily sleep time of children (**<**10 h)**					
10–12 h	0.96 (0.77–1.20)	0.96 (0.74–1.24)	0.86 (0.65–1.15)	0.92 (0.66–1.28)	1.42 (1.15–1.75)	1.11 (0.88–1.41)	0.89 (0.74–1.09)	1.17 (0.93–1.47)
≥12 h	**0.41 (0.30**–**0.56)**	**0.58 (0.38**–**0.87)**	**0.23 (0.14**–**0.38)**	**0.47 (0.25**–**0.88)**	1.31 (1.04–1.65)	0.92 (0.67–1.27)	**0.14 (0.09**–**0.20)**	**0.38 (0.24**–**0.61)**
**Residential floor (1–3 floors)**					
4–6 floors	1.14 (0.90–1.45)	1.04 (0.80–1.34)	1.00 (0.74–1.35)	0.92 (0.66–1.29)	**1.29 (1.04**–**1.61)**	**1.27 (1.01**–**1.60)**	**1.28 (1.04**–**1.58)**	1.16 (0.92–1.47)
≥7 floors	1.26 (0.98–1.62)	1.24 (0.95–1.62)	0.89 (0.64–1.23)	0.85 (0.59–1.22)	1.24 (0.99–1.56)	1.17 (0.92–1.49)	0.96 (0.76–1.20)	1.00 (0.66–1.50)
**Decoration situation of the permanent residence (Never)**								
Before pregnancy	**1.68 (1.15**–**2.45)**	1.35 (0.90–2.01)	1.11 (0.64–1.92)	0.84 (0.46–1.54)	0.81 (0.54–1.22)	**0.65 (0.42**–**0.99)**	1.22 (0.85–1.76)	0.51 (0.25–1.04)
during pregnancy	**1.81 (1.06**–**3.10)**	**1.79 (1.01**–**3.16)**	1.54 (0.76–3.13)	1.27 (0.56–2.85)	1.05 (0.60–1.83)	0.95 (0.53–1.71)	0.61 (0.31–1.19)	1.13 (0.87–1.48)
after birth	**1.43 (1.09**–**1.89)**	1.11 (0.82–1.51)	**1.92 (1.39**–**2.65)**	1.42 (0.98–2.05)	1.12 (0.86–1.45)	1.27 (0.96–1.68)	**1.80 (1.42**–**2.28)**	1.01 (0.81–1.27)
**Wall materials for children's rooms (Others)**					
Wall painting	1.92 (0.74–1.14)	1.06 (0.84–1.35)	0.83 (0.63–1.12)	1.00 (0.72–1.38)	1.20 (0.99–1.46)	**1.31 (1.06**–**1.61)**	0.84 (0.69–1.03)	0.93 (0.61–1.41)
**Habit of keeping windows open in summer (No)**								
Sometimes	0.84 (0.66–1.07)	0.92 (0.68–1.24)	0.99 (0.73–1.34)	1.16 (0.79–1.71)	0.85 (0.68–1.05)	0.94 (0.72–1.23)	1.03 (0.83–1.28)	0.96 (0.69–1.34)
Often	**0.74 (0.58**–**0.94)**	0.89 (0.62–1.28)	0.84 (0.61–1.15)	0.88 (0.55–1.40)	**0.61 (0.49**–**0.77)**	**0.70 (0.50**–**0.98)**	1.06 (0.85–1.31)	1.09 (0.71–1.67)
**Habit of keeping windows open in winter (No)**					
Sometimes	0.80 (0.63–1.02)	1.14 (0.83–1.57)	0.65 (0.47–0.90)	**0.64 (0.42**–**0.98)**	0.98 (0.79–1.21)	1.07 (0.80–1.42)	0.82 (0.66–1.02)	0.80 (0.54–1.20)
Often	**0.62 (0.46**–**0.84)**	1.01 (0.64–1.60)	**0.51 (0.34**–**0.77)**	**0.53 (0.29**–**0.97)**	**0.72 (0.55**–**0.93)**	0.91 (0.61–1.36)	**0.73 (0.56**–**0.94)**	1.06 (0.80–1.41)
**Mother's exposure to secondhand smoke from family members during pregnancy (No)**								
Yes	**1.38 (1.07**–**1.80)**	**1.37 (1.03**–**1.83)**	1.14 (0.80–1.61)	0.91 (0.61–1.34)	1.18 (0.92–1.51)	1.18 (0.90–1.54)	1.12 (0.88–1.43)	0.78 (0.37–1.63)

Group in () Reference category; crude OR was calculated by univariate logistic regression model; Adjusted OR was calculated by multivariable logistic regression model included all independent variables. The bold value indicates statistical significance (*P*<*0.05*).

In terms of sociodemographic characteristics, boys are associated with increased OR of asthma (*AOR* = *1.97, 95% CI: 1.47–2.64*), ethnic minority are associated with increased OR of asthma (*AOR* = *1.85, 95% CI: 1.12–3.04*). Besides, increasing age, treated as a continuous variable, was associated with lower OR of AD (*AOR* = *0.85, 95%CI: 0.80–0.92*) but higher OR of AR (*AOR* = *1.23, 95%CI: 1.14–1.32*). Higher socioeconomic status was associated with increased OR of FA (*AOR* = *3.33, 95%CI: 1.19–9.27*) and AR (*AOR* = *2.49, 95%CI: 1.02–6.05*).

Regarding the maternal exposure during pregnancy and living environment, maternal exposure to secondhand smoke from family members during pregnancy (*AOR* = *1.38, 95%CI: 1.03–1.84*) and residence in a newly renovated room during pregnancy (*AOR* = *1.78, 95%CI: 1.01–3.16*) were associated with higher OR of FA. Window ventilation at least once per day, often use of fresh air ventilation systems, and leaving windows open for ventilation during winter were associated with reduced OR of Asthma with AORs of 0.17 (*95% CI: 0.05–0.61*), 0.34 (*95% CI: 0.14–0.82*), and 0.64 (*95% CI: 0.42–0.98*), respectively. While, residing on floors 4–6 was associated with a increased OR for AD (AOR = 1.27, *95% CI: 1.01–1.60)*. Frequent window ventilation during summer and often use of fresh air ventilation systems was associated with a reduced OR for AR, AOR = 0.70 (*95% CI: 0.50–0.98*) and 0.34 (*95% CI: 0.14–0.82*).

### 3.4 Subgroup and sensitivity analyses

We performed a sensitivity analysis using a multivariable logistic regression model that was additionally adjusted for birth weight and the number of children in the household, the results of this analysis remained largely unchanged from those presented in Table 2 (Supplementary Table S2).

Furthermore, we explored potential effect modification by age and sex through tests of interaction (Wald test). Two significant interactions were identified: first between sex and window ventilation (≥1 time/day) for asthma (*P* = *0.022*); and second, between age (as a continuous variable) and average sleep duration for AR (*P* = *0.001*).

Stratified analyses revealed that the association between window ventilation (≥1 time/day) and reduced OR of Asthma was significant only among boys. For AR, the association of longer sleep duration (>12 h) with reduced odds was observed exclusively in children under 3 years of age with AOR = 0.25 (*95% CI: 0.13–0.48*). (Supplementary Tables S3 and S4).

## 4 Discussion

To our knowledge, the CACSQ represents the first validated instrument designed to concurrently evaluate the four major pediatric allergic conditions—FA, AD, AR), and asthma—specifically tailored for Chinese children aged 0–6 years. Characterized by robust psychometric properties, the CACSQ fills a critical methodological gap in pediatric allergy screening, successfully distinguishing itself from existing screening tools that are constrained to evaluating only one to three atopic diseases (10, 24, 25). The integration of pre-specified diagnostic cutoff values elevates its utility beyond large-scale epidemiological mapping, enabling immediate, practical implementation in primary healthcare and community settings for rapid case identification. Utilizing this newly validated tool, our large-scale cross-sectional survey unveiled a substantial and intricate burden of early-childhood allergies within a modern urban Chinese populace. While we identified a core set of universal determinants—most notably familial predisposition and early-life antibiotic exposure—our modeling further delineated a highly nuanced constellation of shared and distinct environmental and lifestyle influences for individual atopic outcomes. Crucially, the positive association observed between elevated socioeconomic status and specific allergy risks highlights a complex socioeconomic gradient that challenges oversimplified, traditional interpretations of the hygiene hypothesis.

From an implementation standpoint, the instrument exhibits outstanding feasibility, requiring an average completion time of 241 s and yielding high caregiver acceptance. Cronbach's α and Cohen's kappa are widely recognized benchmarks for evaluating internal consistency and test-retest reliability within medical research (26, 27). The acceptable threshold for Cronbach's α typically spans from 0.70 to 0.95 (28), whereas kappa coefficients are conventionally interpreted as follows: ≤ 0 indicates no agreement, 0.01–0.20 slight, 0.21–0.40 fair, 0.41–0.60 moderate, 0.61–0.80 substantial, and 0.81–1.00 almost perfect agreement (29). Comprising 24 items, the CACSQ demonstrated excellent internal consistency (Cronbach's α: 0.85–0.89), with its test-retest reliability reflecting substantial to almost perfect concordance (Kappa: 0.68–0.88). Collectively, these robust psychometric properties substantiate the high reliability and validity of the instrument, strongly supporting its suitability for assessing pediatric allergic disease states.

Our findings are consistent with most previous studies in which cesarean section, only one child in the household and exposure to second-hand smoke were associated with an increased risk of allergic diseases among children (20, 30–32). The observed association between higher SES (proxied by parental education) and increased allergy risk resonates with global patterns often described as the allergy epidemic associated with economic development (33, 34). This phenomenon may be mediated by a constellation of factors linked to improved living conditions, including increased time spent indoors, dietary changes, and the characteristics of the gut microbiota in the early stage of life (35, 36). Our assessment of dwelling environment exposure allowed us to probe specific elements of this complex picture. For instance, the inverse association of frequent window ventilation and fresh air system use against Asthma and AR provides tangible support for the role of improved indoor air quality and dilution of airborne allergens or volatile organic compounds. As reviewed by Eguiluz-Gracia et al., (37) indoor and outdoor air pollution, along with the ensuing climate change, significantly influence the development and severity of allergic rhinitis and asthma in both children and adults . In parallel, Huang et al., (38) also identified indoor air quality and specific household environmental characteristics as potential factors associated with the development and exacerbation of allergic diseases among preschool children.

Our findings indicate that preterm birth is associated with a significantly elevated risk of allergic rhinitis, corroborating previous literature (39). The underlying pathophysiological mechanism may involve immature lung development, heightened airway hyperresponsiveness, and early-childhood immune dysregulation (40). Furthermore, early-life antibiotic exposure has been heavily implicated in pediatric atopy; for instance, Mitselou et al. (39) and Lu et al. (41) identified a link between early childhood antibiotic use and increased food allergy risks, while a retrospective cohort study by Beier et al. (42) demonstrated that early-life antibiotic contact positively correlates with asthma, food allergy, and allergic rhinitis, displaying a dose-response relationship with multiple courses. Echoing these established patterns, our study revealed that a history of otitis media and a history of antibiotic treatment were concurrently tied to elevated risks across all four conditions (food allergy, asthma, atopic dermatitis, and allergic rhinitis), whereas a history of pneumonia uniquely predisposed children to food allergy, asthma, and allergic rhinitis. This vulnerability is likely attributable to the disruption of early microbial colonization by antibiotic interventions, which precipitates gut dysbiosis and subsequently primes the host for allergies and asthma (43). While these findings align with the hypothesis that early microbial disruption may precipitate gut dysbiosis and immune skewing, they must be interpreted with caution. Due to the cross-sectional design, these associations may also reflect reverse causation, increased healthcare utilization, or a shared underlying susceptibility to both respiratory infections and allergic inflammation, rather than a direct causal pathway. Beyond atopic outcomes, the potential correlations between maternal or early-infant antibiotic exposure and heightened risks of autoimmune conditions and neurodevelopmental disorders—such as autism spectrum disorder and intellectual disability—are highly noteworthy and warrant serious consideration (44, 45).

The intricate patterns of comorbidity among asthma, FA, AD, and AR, coupled with their partially overlapping yet distinct environmental triggers, reflect the concept of the “atopic march” (46). Our data suggest that early-life exposures may program the immune system toward a Th2-skewed phenotype (47), which then manifests as different conditions along a developmental trajectory (48). The present study found disease-specific associations with early life health factors, providing important epidemiological evidence for this hypothesis. For example, in this study, cesarean section was specifically associated with a higher risk of atopic dermatitis, and this association may be mediated through effects on the infant gut microbiome. Further supporting evidence comes from the studies by Mubanga et al. (49) and Marrs et al. (50); the former reported, based on a large cohort, that children delivered by cesarean section or instrumental vaginal delivery had a higher risk of early-onset atopic dermatitis, whereas the latter, using a randomized controlled trial design, analyzed the gut microbiota profiles of three-month-old breastfed infants and found that cesarean section was associated with a distinct gut microbial signature (increased abundance of Clostridium perfringens in children born by cesarean section, a bacterium closely linked to the pathogenesis of atopic dermatitis).

Additionally, our findings demonstrate that an outdoor physical activity duration of ≥ 2 h/day is inversely associated with childhood allergic rhinitis. Concurrently, a daily sleep duration of ≥ 12 h exhibited a significant inverse association with food allergy, asthma, and allergic rhinitis, a discovery that aligns with a growing body of evidence highlighting the immunomodulatory role of sleep. Sleep and the immune system share a bidirectional relationship: whereas immune activation can alter sleep architecture and patterns, sufficient sleep reinforces immune defenses by mitigating infection risks and optimizing vaccine responsiveness. Conversely, chronic sleep deprivation disrupts this delicate equilibrium, thereby fueling systemic low-grade inflammation and elevating susceptibility to a spectrum of inflammatory disorders (51–53). This intricate interplay between sleep and atopic conditions is conversely corroborated by Esteban et al. (54), who demonstrated that among urban, school-aged children with asthma, a higher burden of positive allergy tests (atopic sensitization) was significantly coupled with objectively measured poorer sleep outcomes.

Notably, our study identified significant interaction effects that warrant attention. The inverse association of daily window ventilation against asthma was observed predominantly in boys, which may be attributed to sex-based differences in early-life airway caliber or distinct indoor activity patterns, making boys more susceptible to indoor allergen accumulation (55). Additionally, the inverse association between prolonged sleep duration and allergic rhinitis was exclusively significant in children under 3 years of age. This highlights a critical developmental window wherein sufficient sleep may play a profound immunomodulatory role, actively shaping early immune tolerance before the complete maturation of the pediatric immune system (51, 56).

There are four important strengths in this study. First, the rigorous multistage, multilevel random sampling method ensured a highly representative sample. Second, the high response rate minimized potential selection bias. Third, the investigation encompassed multiple childhood allergic conditions—including asthma, allergic rhinitis, food allergy, and atopic dermatitis—enabling a comprehensive assessment of the associations of perinatal factors, living environment, family background, and dietary patterns with these diseases. Finally, we developed and validated the CACSQ, which demonstrates comparable or superior performance to established tools (24, 25, 57, 58). It provides a standardized, low-cost, and efficient tool for large-scale epidemiological studies and initial community-based screening in resource-limited settings.

The study has several limitations. First, all information was obtained from questionnaires, which inevitably introduces some degree of bias (e.g., recall bias and measurement bias). Furthermore, while our expert consultation process achieved high consensus, we did not calculate formal quantitative Content Validity Indices (CVI) during scale development, which should be considered when interpreting the psychometric outcomes. And because the optimal diagnostic cut-offs and the questionnaire's performance metrics were derived and evaluated within the same validation sample, there is a possibility of optimistic estimation. Future studies employing independent external validation cohorts are required to confirm these diagnostic thresholds. While we adjusted for a wide array of confounders, residual confounding by unmeasured factors, such as precise outdoor air pollution exposure, remains possible. The cross-sectional design precludes causal inference, and the possibility of reverse causality cannot be ruled out. Nevertheless, factors such as the child's age, being an only child, and family history of allergies were already significantly present before the diagnosis of allergic diseases, thus establishing a temporal sequence.

## 5 Conclusion

In conclusion, childhood allergic diseases affect a high proportion of preschool-aged children in urban China. The newly developed CACSQ is a valid, reliable, and highly efficient multi-disease screening tool suitable for pediatric populations aged 0–6 years.

Our cross-sectional survey shows that while genetic predisposition remains a strong risk factor, modifiable modern lifestyle and environmental factors—such as ensuring longer sleep duration, avoiding early-life antibiotic overuse, and maintaining frequent indoor window-opening ventilation—are associated with lower odds of allergic outcomes. While the CACSQ demonstrates strong potential, further multi-center validation in geographically and socioeconomically diverse populations is warranted before considering its routine implementation in broad primary care settings.

## Data Availability

The raw data supporting the conclusions of this article will be made available by the authors, without undue reservation.
