# Supplementary material for: Development and validation of the childhood allergy comprehensive screening questionnaire and Its application in a cross-sectional survey of allergic diseases among children aged 0–6 Years in Shanghai, China
Source: Front Public Health. 2026 Jul 29;14:1906537. doi: 10.3389/fpubh.2026.1906537 (PMC13462234; doi:10.3389/fpubh.2026.1906537)
Supplement: Supplementary file 1 [file Supplementary_file_1.docx]

S1. Comorbidity of asthma and childhood allergic diseases

|  | Total (%) | Sex (%) | | P-Value | Age (%) | | P-Value |
| --- | --- | --- | --- | --- | --- | --- | --- |
|  | 3064 | girl | boy | 0.586 | ＜3 year | ≥3 year | ＜0.001 |
| Food allergic | 451 (14.7%) | 217 (48.1%) | 234 (51.9%) |  | 129 (28.6%) | 322 (71.4%) |  |
|  | | | | ＜0.001 |  |  | ＜0.001 |
| Asthma | 256 (8.4%) | 86 (33.6%) | 170 (66.4%) |  | 46 (18.0%) | 210 (82.0%) |  |
|  | | | | 0.946 |  |  | 0.001 |
| Atopic dermatitis | 578 (18.9%) | 272 (47.1%) | 306 (52.9%) |  | 274 (47.4%) | 304 (52.6%) |  |
|  | | | | 0.573 |  |  | ＜0.001 |
| Allergic rhinitis | 612 (20.0%) | 281 (45.9%) | 331 (54.1%) |  | 102 (16.7%) | 510 (83.3%) |  |
| Coexisting | | | | 0.057 |  |  | ＜0.001 |
| Food allergic and asthma | 70 (2.3%) | 25 (35.7%) | 45 (64.3%) |  | 10 (14.3%) | 60 (85.7%) |  |
|  | | | | 0.424 |  |  | 0.444 |
| Food allergic and atopic dermatitis | 160 (5.2%) | 80 (50.0%) | 80 (50.0%) |  | 61 (38.1%) | 99 (61.9%) |  |
|  | | | | 0.544 |  |  | ＜0.001 |
| Food allergic and allergic rhinitis | 181 (5.9%) | 81 (44.8%) | 100 (55.2%) |  | 25 (13.8%) | 156 (86.2%) |  |
|  | | | | 0.021 |  |  | ＜0.001 |
| Asthma and atopic dermatitis | 79 (2.6%) | 27 (34.2%) | 52 (65.8%) |  | 12 (15.2%) | 67 (84.8%) |  |
|  | | | | 0.003 |  |  | ＜0.001 |
| Asthma and allergic rhinitis | 129 (4.2%) | 44 (34.1%) | 85 (65.9%) |  | 16 (12.4%) | 113 (87.6%) |  |
|  | | | | 0.999 |  |  | ＜0.001 |
| Atopic dermatitis and allergic rhinitis | 179 (5.8%) | 84 (46.9%) | 95 (53.1%) |  | 41 (22.9%) | 138 (77.1%) |  |
|  | | | | 0.050 |  |  | ＜0.001 |
| Asthma and Atopic dermatitis and allergic rhinitis | 46 (1.5%) | 15 (32.6%) | 31 (67.4%) |  | 4 (8.7%) | 42 (91.3%) |  |
|  | | | | 0.598 |  |  | ＜0.001 |
| Food allergic, atopic dermatitis and allergic rhinitis | 72 (2.3%) | 36 (50.0%) | 36 (50.0%) |  | 15 (20.8%) | 57 (79.2%) |  |
|  | | | | 0.172 |  |  | ＜0.001 |
| Food allergic, asthma and allergic rhinitis | 46 (1.5%) | 17 (37.0%) | 29 (63.0%) |  | 3 (6.5%) | 43 (93.5%) |  |
|  | | | | 0.575 |  |  | ＜0.001 |
| Food allergic, asthma and atopic dermatitis | 31 (1.0%) | 13 (41.9%) | 18 (58.1%) |  | 3 (9.7%) | 28 (90.3%) |  |
|  | | | | 0.707 |  |  | ＜0.001 |
| Comorbid for all four conditions | 21 (0.7%) | 9 (42.9%) | 12 (57.1%) |  | 0 (0.0%) | 21 (100.0%) |  |

Data were presented with frequency and percentage; *p*-value was calculated by Pearson’s chi-squared (χ2) test;

S2. Sensitivity Analysis: Multivariable Logistic Regression for Childhood Allergies After Adjusting for Birth Weight and Number of Children

| **Variable** | **Food allergy (AOR)** | **Asthma (AOR)** | **Atopic dermatitis (AOR)** | **Allergic rhinitis (AOR)** |
| --- | --- | --- | --- | --- |
| Age | 1.01 (0.94-1.09) | 1.03 (0.92-1.14) | **0.85 (0.79-0.91)** | **1.22 (1.13-1.31)** |
| Number of children | 0.87 (0.71-1.06) | 1.11 (0.87-1.43) | 0.88 (0.74-1.05) | **0.79 (0.66-0.95)** |
| air conditioners usage/months | **1.07 (1.02-1.13)** | **1.09 (1.02-1.18)** | **1.06 (1.01-1.12)** | **1.08 (1.03-1.14)** |
| Gender (girls) | | | | |
| boys | 0.95 (0.77-1.17) | **1.96 (1.47-2.63)** | 0.99 (0.82-1.20) | 1.06 (0.87-1.29) |
| race (Han) | | | | |
| ethnic minority | **1.68 (1.00-2.80)** | 1.10 (0.54-2.26) | 1.07 (0.63-1.80) | 0.57 (0.31-1.07) |
| Degree of mother (Junior high school and below) | | | | |
| High/vocational high school | 1.21 (0.42-3.49) | 1.62 (0.46-5.68) | 0.60 (0.32-1.14) | 0.87 (0.37-2.05) |
| Undergraduate | **3.00 (1.13-8.00)** | 1.75 (0.52-5.89) | 0.60 (0.33-1.08) | 1.19 (0.53-2.63) |
| Postgraduate | **3.26 (1.18-9.05)** | 2.15 (0.60-7.70) | 0.62 (0.33-1.18) | 1.14 (0.49-2.64) |
| Degree of Father (Junior high school and below) | | | | |
| High/vocational high school | 0.61 (0.25-1.45) | 0.87 (0.29-2.67) | 1.27 (0.64-2.53) | 1.38 (0.56-3.36) |
| Undergraduate | 0.69 (0.31-1.53) | 0.97 (0.34-2.76) | 1.78 (0.93-3.42) | 2.20 (0.95-5.09) |
| Postgraduate | 0.71 (0.31-1.65) | 0.69 (0.23-2.11) | 1.78 (0.88-3.58) | 2.35 (0.98-5.67) |
| Premature birth (No) | | | | |
| Yes | 1.46 (0.97-2.20) | 1.13 (0.68-1.88) | 0.97 (0.71-1.34) | **1.57 (1.06-2.32)** |
| Birth mode (Vaginal delivery) | | | | |
| Cesarean section | 1.10 (0.88-1.36) | 0.89 (0.67-1.18) | 0.85 (0.70-1.03) | 0.94 (0.77-1.15) |
| Has the child ever had pneumonia (No) | | | | |
| Yes | **1.62 (1.28-2.06)** | **3.65 (2.71-4.92)** | 1.08 (0.86-1.37) | **1.55 (1.25-1.92)** |
| Has the child ever had Pediatric inflammation (No) | | | | |
| Yes | 1.23 (0.83-1.81) | 1.21 (0.75-1.94) | **1.69 (1.18-2.43)** | 1.34 (0.95-1.89) |
| Has the child ever received antibiotic treatment (No) | | | | |
| Yes | **1.38 (1.04-1.82)** | **2.60 (1.65-4.11)** | **1.32 (1.03-1.68)** | **1.67 (1.29-2.17)** |
| Family historyof allergy (No) | | | | |
| Yes | **2.30 (1.82-2.90)** | **2.16 (1.57-2.96)** | **2.35 (1.92-2.89)** | **2.84 (2.28-3.52)** |
| Fresh air systems usage frequency (No) | | | | |
| Sometimes | 0.86 (0.60-1.24) | 1.20 (0.76-1.91) | 0.85 (0.61-1.19) | **0.69 (0.48-0.98)** |
| Often | 1.19 (0.75-1.88) | **0.33 (0.14-0.82)** | 0.67 (0.42-1.05) | 0.77 (0.48-1.23) |
| Incense/air fresheners use (No) | | | | |
| Sometimes | 0.91 (0.67-1.23) | 0.99 (0.67-1.46) | 0.94 (0.70-1.26) | 1.13 (0.86-1.48) |
| Often | 0.97 (0.51-1.84) | 0.73 (0.31-1.76) | 0.83 (0.45-1.50) | **1.95 (1.15-3.29)** |
| Open the Windows for ventilation within one day (0 times) | | | | |
| 1 | 0.45 (0.13-1.47) | **0.17 (0.05-0.61)** | **0.34 (0.12-0.95)** | 0.32 (0.09-1.14) |
| 2 | 0.55 (0.17-1.83) | **0.15 (0.04-0.54)** | **0.33 (0.12-0.92)** | 0.41 (0.12-1.43) |
| ≥3 | 0.46 (0.14-1.51) | **0.10 (0.03-0.36)** | **0.34 (0.12-0.94)** | 0.41 (0.12-1.42) |
| Daily exercise time for children (＜ 2 hours) | | | | |
| ≥2h | 1.08 (0.87-1.35) | 0.90 (0.67-1.21) | 1.01 (0.83-1.24) | 0.83 (0.68-1.02) |
| daily sleep time of children (＜10 hours) | | | | |
| 10-12 h | 0.96 (0.75-1.24) | 0.92 (0.66-1.28) | 1.11 (0.88-1.41) | 1.17 (0.93-1.47) |
| ≥12 h | **0.57 (0.38-0.87)** | **0.46 (0.25-0.88)** | 0.92 (0.67-1.26) | **0.38 (0.23-0.60)** |
| Residential floor (1-3 floors) | | | | |
| 4-6 floors | 1.04 (0.80-1.34) | 0.92 (0.66-1.28) | **1.27 (1.01-1.60)** | 1.16 (0.92-1.46) |
| ≥7 floors | 1.24 (0.95-1.62) | 0.85 (0.60-1.22) | 1.18 (0.92-1.50) | 0.99 (0.77-1.28) |
| Decoration situation of the permanent residence (Never) | | | | |
| Before pregnancy | 1.35 (0.90-2.02) | 0.84 (0.46-1.53) | **0.65 (0.42-1.00)** | 1.00 (0.66-1.50) |
| during pregnancy | **1.78 (1.01-3.16)** | 1.26 (0.56-2.84) | 0.96 (0.53-1.71) | 0.51 (0.25-1.04) |
| after birth | 1.12 (0.83-1.51) | 1.41 (0.97-2.04) | 1.27 (0.96-1.68) | 1.14 (0.87-1.49) |
| Wall materials for children's rooms (Others) | | | | |
| wall painting | 1.06 (0.84-1.35) | 1.01 (0.73-1.39) | **1.30 (1.06-1.61)** | 1.01 (0.81-1.27) |
| Habit of keeping Windows open in summer (No) | | | | |
| Sometimes | 0.92 (0.68-1.24) | 1.16 (0.79-1.71) | 0.94 (0.72-1.23) | 1.07 (0.81-1.42) |
| Often | 0.88 (0.61-1.27) | 0.87 (0.54-1.39) | **0.69 (0.49-0.97)** | 0.95 (0.68-1.32) |
| Habit of keeping Windows open in winter (No) | | | | |
| Sometimes | 1.14 (0.83-1.57) | **0.65 (0.43-0.99)** | 1.07 (0.80-1.42) | 0.98 (0.73-1.31) |
| Often | 1.02 (0.65-1.61) | **0.53 (0.29-0.97)** | 0.92 (0.61-1.37) | 0.82 (0.55-1.22) |
| Mother's exposure to secondhand smoke from family members during pregnancy (No) | | | | |
| Yes | **1.38 (1.03-1.83)** | 0.91 (0.61-1.34) | 1.18 (0.90-1.54) | 1.06 (0.80-1.40) |

Data were presented with OR and 95% CI; AOR adjusted odds ratio, CI confidence interval

The bold value indicates statistical significance (P < 0.05)

S3. Stratified Analysis: Multivariable Logistic Regression Results for Childhood Allergies and Independent Variables by Sex.

| Variable | Food allergy (AOR) | | Asthma (AOR) | | Atopic dermatitis (AOR) | | Allergic rhinitis (AOR) | |
| --- | --- | --- | --- | --- | --- | --- | --- | --- |
|  | girl | boy | girl | boy | girl | boy | girl | boy |
| Age | 1.00 (0.89-1.12) | 1.04 (0.93-1.16) | 1.03 (0.86-1.24) | 1.00 (0.88-1.15) | **0.88 (0.79-0.98)** | **0.83 (0.75-0.91)** | **1.24 (1.11-1.38)** | **1.24 (1.12-1.38)** |
| air conditioners usage/months | 1.01 (0.93-1.09) | **1.14 (1.06-1.23)** | 1.09 (0.96-1.24) | **1.12 (1.03-1.23)** | **1.09 (1.01-1.17)** | 1.03 (0.96-1.10) | 1.04 (0.96-1.12) | **1.12 (1.04-1.21)** |
| Race (Han) | | | | | | | | |
| ethnic minority | 1.92 (0.90-4.07) | 1.51 (0.73-3.15) | 0.81 (0.24-2.71) | 1.02 (0.40-2.63) | 0.51 (0.21-1.28) | 1.62 (0.83-3.15) | **0.20 (0.06-0.69)** | 1.05 (0.49-2.25) |
| Number of children (1) | | | | | | | | |
| 2 | 0.79 (0.56-1.12) | 0.79 (0.57-1.11) | 1.45 (0.86-2.46) | 1.02 (0.68-1.51) | 0.87 (0.64-1.20) | 0.77 (0.57-1.04) | **0.68 (0.49-0.94)** | **0.68 (0.50-0.93)** |
| 3 | 1.23 (0.47-3.18) | 1.06 (0.46-2.46) | 0.96 (0.17-5.40) | 1.56 (0.65-3.74) | 0.80 (0.33-1.93) | 1.29 (0.66-2.50) | 1.19 (0.51-2.79) | 1.33 (0.62-2.85) |
| ≥4 | 2.00 (0.15-27.60) | 0.83 (0.01-7.95) | 0.84 (0.01-14.22) | 2.43 (0.21-28.68) | 0.98 (0.09-10.88) | 0.29 (0.00-2.58) | 0.42 (0.00-7.23) | 1.05 (0.10-11.27) |
| Degree of mother (Junior high school and below) | | | | | | | | |
| High school/vocational high school | 1.11 (0.27-4.64) | 1.32 (0.24-7.42) | 0.97 (0.11-8.29) | 2.34 (0.41-13.21) | 0.44 (0.18-1.08) | 0.83 (0.31-2.24) | 1.13 (0.31-4.17) | 0.57 (0.17-1.92) |
| Undergraduate | 2.78 (0.73-10.50) | 3.34 (0.66-16.80) | 2.12 (0.27-16.50) | 1.85 (0.35-9.93) | 0.52 (0.23-1.15) | 0.77 (0.30-2.02) | 1.35 (0.40-4.52) | 0.95 (0.31-2.92) |
| Postgraduate | 3.26 (0.81-13.14) | 3.26 (0.62-17.16) | 1.93 (0.22-16.83) | 2.50 (0.44-14.36) | 0.50 (0.21-1.22) | 0.87 (0.31-2.42) | 1.47 (0.41-5.27) | 0.83 (0.26-2.72) |
| Degree of Father (Junior high school and below) | | | | | | | | |
| High/vocational high school | 0.58 (0.15-2.17) | 0.79 (0.23-2.76) | 0.59 (0.08-4.41) | 1.31 (0.29-5.90) | 1.16 (0.43-3.16) | 1.34 (0.48-3.77) | 5.08 (0.95-27.31) | 0.75 (0.23-2.40) |
| Undergraduate | 0.75 (0.23-2.48) | 0.84 (0.27-2.63) | 0.54 (0.08-3.69) | 1.44 (0.35-5.89) | 1.60 (0.65-3.95) | 1.95 (0.72-5.28) | 5.17 (0.99-26.90) | 1.85 (0.65-5.30) |
| Postgraduate | 0.80 (0.23-2.84) | 0.88 (0.26-2.92) | 0.40 (0.05-3.09) | 1.00 (0.23-4.44) | 1.40 (0.52-3.74) | 2.07 (0.72-5.95) | **6.47 (1.19-35.30)** | 1.77 (0.58-5.39) |
| Premature birth (No) | | | | | | | | |
| Yes | 1.89 (0.97-3.71) | 1.28 (0.75-2.18) | 2.97 (0.79-11.11) | 0.96 (0.54-1.70) | 1.15 (0.70-1.89) | 0.96 (0.64-1.45) | 0.99 (0.57-1.72) | **2.44 (1.39-4.28)** |
| Birth mode (Vaginal delivery) | | | | | | | | |
| Cesarean section | 1.00 (0.73-1.37) | 1.25 (0.92-1.69) | 1.14 (0.69-1.88) | 0.76 (0.53-1.09) | 0.99 (0.75-1.32) | **0.73 (0.56-0.96)** | 0.94 (0.70-1.26) | 0.90 (0.68-1.20) |
| Has the child ever had pneumonia (No) | | | | | | | | |
| Yes | **1.75 (1.23-2.48)** | **1.54 (1.10-2.16)** | **2.95 (1.76-4.95)** | **4.44 (3.01-6.55)** | 1.07 (0.75-1.52) | 1.08 (0.78-1.49) | 1.32 (0.95-1.83) | **1.85 (1.37-2.50)** |
| Has the child ever had Pediatric inflammation (No) | | | | | | | | |
| Yes | 1.27 (0.72-2.25) | 1.20 (0.69-2.09) | 1.38 (0.61-3.12) | 1.20 (0.64-2.26) | 1.31 (0.74-2.29) | **2.03 (1.24-3.34)** | 1.46 (0.86-2.47) | 1.24 (0.77-2.00) |
| Has the child ever received antibiotic treatment (No) | | | | | | | | |
| Yes | **1.64 (1.10-2.45)** | 1.20 (0.80-1.79) | **3.24 (1.50-7.01)** | **2.52 (1.39-4.56)** | 1.34 (0.94-1.90) | 1.37 (0.96-1.95) | **1.98 (1.35-2.88)** | 1.44 (0.99-2.10) |
| Family historyof allergy (No) | | | | | | | | |
| Yes | **2.03 (1.45-2.85)** | **2.58 (1.84-3.61)** | 1.52 (0.88-2.61) | **2.54 (1.69-3.80)** | **2.56 (1.88-3.48)** | **2.33 (1.75-3.10)** | **2.68 (1.94-3.71)** | **3.10 (2.29-4.20)** |
| Fresh air systems use (No) | | | | | | | | |
| Sometimes | 0.99 (0.59-1.68) | 0.79 (0.46-1.36) | 1.31 (0.59-2.94) | 1.19 (0.65-2.18) | 0.96 (0.59-1.58) | 0.80 (0.49-1.30) | 0.71 (0.42-1.21) | 0.72 (0.44-1.20) |
| Often | 1.28 (0.67-2.42) | 1.21 (0.61-2.40) | 0.41 (0.09-1.86) | **0.27 (0.09-0.85)** | 0.71 (0.37-1.37) | 0.61 (0.32-1.16) | 1.04 (0.54-1.97) | 0.60 (0.29-1.24) |
| Incense/air fresheners use (No) | | | | | | | | |
| Sometimes | 0.90 (0.58-1.42) | 0.86 (0.55-1.33) | 0.62 (0.30-1.29) | 1.34 (0.81-2.21) | 0.82 (0.53-1.27) | 1.02 (0.68-1.53) | 1.10 (0.73-1.66) | 1.07 (0.73-1.59) |
| Often | 1.03 (0.41-2.57) | 0.85 (0.33-2.16) | 1.15 (0.33-4.01) | 0.49 (0.14-1.80) | 0.82 (0.32-2.09) | 0.90 (0.41-2.01) | 1.91 (0.87-4.20) | **2.20 (1.04-4.65)** |
| Open the Windows for ventilation within one day (0 times) | | | | | | | | |
| 1 | 0.45 (0.08-2.68) | 0.41 (0.07-2.22) | 0.93 (0.06-14.07) | **0.07 (0.01-0.37)** | 0.28 (0.05-1.46) | 0.38 (0.09-1.56) | 0.29 (0.04-1.97) | 0.23 (0.04-1.37) |
| 2 | 0.33 (0.06-1.97) | 0.84 (0.16-4.49) | 0.34 (0.02-5.24) | **0.11 (0.02-0.54)** | 0.25 (0.05-1.28) | 0.39 (0.10-1.59) | 0.33 (0.05-2.24) | 0.34 (0.06-1.95) |
| ≥3 | 0.38 (0.07-2.18) | 0.55 (0.10-2.95) | 0.38 (0.03-5.55) | **0.06 (0.01-0.29)** | 0.30 (0.06-1.50) | 0.38 (0.09-1.54) | 0.32 (0.05-2.16) | 0.33 (0.06-1.90) |
| Daily exercise time for children (＜ 2 hours) | | | | | | | | |
| ≥2h | **1.42 (1.02-1.95)** | 0.87 (0.63-1.19) | 0.89 (0.53-1.48) | 0.86 (0.59-1.25) | 1.18 (0.87-1.60) | 0.90 (0.68-1.19) | 0.82 (0.60-1.12) | 0.84 (0.63-1.11) |
| Daily sleep time of children (＜10 hours) | | | | | | | | |
| 10-12 h | 0.92 (0.63-1.34) | 1.05 (0.73-1.51) | 1.11 (0.62-1.99) | 0.92 (0.60-1.42) | 1.41 (1.00-1.99) | 0.91 (0.65-1.27) | 0.91 (0.64-1.28) | **1.48 (1.07-2.05)** |
| ≥12 h | 0.66 (0.36-1.19) | **0.51 (0.28-0.93)** | 0.23 (0.05-1.09) | 0.58 (0.28-1.23) | 0.99 (0.61-1.60) | 0.88 (0.56-1.38) | **0.35 (0.17-0.71)** | **0.41 (0.21-0.78)** |
| Are environment near the residence close to rivers, lakes, parks and green Spaces (No) | | | | | | | | |
| Yes | 1.02 (0.58-1.81) | 1.25 (0.69-2.26) | 1.46 (0.63-3.40) | 1.40 (0.69-2.86) | 1.02 (0.62-1.70) | 0.87 (0.50-1.50) | 0.76 (0.42-1.39) | 0.82 (0.45-1.49) |
| Residential floor (1-3 floors) | | | | | | | | |
| 4-6 floors | 1.01 (0.69-1.47) | 1.00 (0.69-1.45) | 1.01 (0.59-1.74) | 0.83 (0.54-1.29) | 1.21 (0.86-1.71) | 1.28 (0.93-1.76) | 1.05 (0.74-1.48) | 1.31 (0.94-1.82) |
| ≥7 floors | 1.31 (0.88-1.93) | 1.20 (0.82-1.75) | **0.40 (0.20-0.82)** | 1.12 (0.72-1.77) | 1.28 (0.89-1.82) | 1.12 (0.79-1.57) | 0.88 (0.60-1.29) | 1.07 (0.75-1.53) |
| Decoration situation of the permanent residence (Never) | | | | | | | | |
| Before pregnancy | 0.88 (0.46-1.68) | **1.81 (1.05-3.09)** | 0.90 (0.31-2.60) | 0.70 (0.32-1.54) | 0.84 (0.46-1.53) | **0.48 (0.26-0.91)** | 1.02 (0.56-1.88) | 0.91 (0.51-1.61) |
| during pregnancy | 1.55 (0.66-3.63) | 2.09 (0.93-4.72) | 1.04 (0.25-4.31) | 1.16 (0.41-3.28) | 0.97 (0.42-2.22) | 1.02 (0.44-2.39) | 0.58 (0.21-1.63) | 0.44 (0.16-1.23) |
| after birth | 0.99 (0.63-1.55) | 1.17 (0.77-1.79) | 1.74 (0.94-3.21) | 1.22 (0.75-1.99) | 1.26 (0.83-1.91) | 1.26 (0.85-1.87) | 1.36 (0.92-2.01) | 0.98 (0.67-1.44) |
| Wall materials for children's rooms (Others) | | | | | | | | |
| wall painting | 0.99 (0.70-1.41) | 1.13 (0.81-1.59) | 0.95 (0.54-1.66) | 1.00 (0.66-1.52) | 1.28 (0.94-1.74) | **1.37 (1.02-1.84)** | 0.98 (0.70-1.37) | 1.06 (0.78-1.46) |
| Habit of keeping Windows open in summer (No) | | | | | | | | |
| Sometimes | 0.89 (0.58-1.38) | 0.90 (0.58-1.39) | 1.34 (0.69-2.61) | 1.09 (0.66-1.82) | 0.97 (0.65-1.45) | 0.89 (0.61-1.32) | 1.27 (0.84-1.93) | 1.02 (0.68-1.52) |
| Often | 0.73 (0.42-1.25) | 1.03 (0.62-1.71) | 1.06 (0.47-2.41) | 0.82 (0.45-1.49) | 0.61 (0.36-1.04) | 0.77 (0.48-1.22) | 0.92 (0.55-1.52) | 0.97 (0.61-1.54) |
| Often | 0.72 (0.27-1.87) | 0.78 (0.32-1.90) | 1.69 (0.40-7.14) | 0.93 (0.34-2.58) | 1.10 (0.46-2.61) | 0.69 (0.31-1.55) | 1.37 (0.54-3.52) | 1.05 (0.47-2.37) |
| Habit of keeping Windows open in winter (No) | | | | | | | | |
| Sometimes | 1.10 (0.69-1.76) | 1.23 (0.78-1.93) | 0.97 (0.47-2.01) | **0.49 (0.28-0.84)** | 1.09 (0.71-1.66) | 1.02 (0.68-1.51) | 0.67 (0.43-1.05) | 1.24 (0.83-1.87) |
| Often | 0.72 (0.36-1.44) | 1.40 (0.75-2.63) | 0.57 (0.17-1.86) | **0.44 (0.21-0.90)** | 1.40 (0.75-2.60) | 0.62 (0.36-1.08) | 0.86 (0.46-1.58) | 0.77 (0.44-1.33) |
| Mother's exposure to secondhand smoke from family members during pregnancy (No) | | | | | | | | |
| Yes | 1.43 (0.94-2.18) | 1.23 (0.81-1.87) | 1.02 (0.52-2.01) | 0.90 (0.54-1.50) | 1.41 (0.97-2.06) | 0.97 (0.65-1.46) | 1.08 (0.71-1.64) | 1.07 (0.71-1.59) |

Data were presented with OR and 95% CI; AOR adjusted odds ratio

Italic value indicates statistical significance (P < 0.05)

S4. Stratified Analysis: Multivariable Logistic Regression Results for Childhood Allergies and Independent Variables by Age.

| Variable | Food allergy (AOR) | | Asthma (AOR) | | Atopic dermatitis (AOR) | | Allergic rhinitis (AOR) | |
| --- | --- | --- | --- | --- | --- | --- | --- | --- |
|  | ＜3 years | ≥3 years | ＜3 years | ≥3 years | ＜3 years | ≥3 years | ＜3 years | ≥3 years |
| air conditioners usage/months | 1.03 (0.94-1.14) | **1.10 (1.03-1.18)** | **1.21 (1.03-1.42)** | 1.07 (0.98-1.16) | 1.07 (1.00-1.14) | 1.06 (0.99-1.13) | 1.12 (1.00-1.25) | 1.05 (0.99-1.12) |
| Gender (Girls) | | | | | | | | |
| Boys | 0.92 (0.62-1.37) | 0.96 (0.74-1.24) | **3.54 (1.60-7.83)** | **1.72 (1.24-2.38)** | 1.08 (0.81-1.45) | 0.92 (0.71-1.19) | 1.05 (0.66-1.68) | 1.08 (0.86-1.35) |
| Race (Han) | | | | | | | | |
| ethnic minority | **2.85 (1.15-7.05)** | 1.38 (0.73-2.61) | 0.71 (0.05-9.37) | 1.11 (0.52-2.41) | 0.84 (0.35-2.01) | 1.19 (0.61-2.33) | 0.20 (0.00-1.46) | 0.68 (0.36-1.30) |
| Number of children (1) | | | | | | | | |
| 2 | 0.94 (0.59-1.51) | **0.74 (0.56-0.98)** | 1.69 (0.76-3.77) | 0.92 (0.65-1.29) | 0.87 (0.62-1.22) | **0.74 (0.56-0.99)** | **0.52 (0.28-0.94)** | **0.71 (0.56-0.90)** |
| 3 | 1.28 (0.51-3.18) | 0.85 (0.35-2.06) | 0.86 (0.14-5.23) | 1.97 (0.83-4.67) | 1.12 (0.57-2.20) | 1.00 (0.44-2.31) | 1.20 (0.41-3.53) | 1.26 (0.63-2.53) |
| ≥4 | 0.53 (0.00-7.48) | 1.18 (0.11-12.63) | **35.95 (1.51-854.57)** | 0.44 (0.00-5.05) | 0.64 (0.07-6.05) | 0.32 (0.00-3.39) | 1.10 (0.01-14.85) | 0.49 (0.04-5.34) |
| Degree of mother (Junior high school and below) | | | | | | | | |
| High/vocational high school | 1.10 (0.28-4.32) | 2.50 (0.28-22.23) | 0.69 (0.08-6.05) | 2.33 (0.43-12.68) | 0.51 (0.23-1.13) | 1.15 (0.32-4.14) | 0.61 (0.08-4.83) | 0.83 (0.31-2.24) |
| Undergraduate | 2.08 (0.58-7.43) | **8.26 (1.02-67.11)** | 0.61 (0.08-4.46) | 2.44 (0.47-12.72) | **0.46 (0.22-0.95)** | 1.14 (0.34-3.81) | 1.51 (0.25-9.33) | 0.94 (0.37-2.38) |
| Postgraduate | 2.86 (0.72-11.40) | 8.25 (0.99-68.94) | 0.89 (0.10-8.23) | 2.84 (0.51-15.72) | 0.45 (0.19-1.03) | 1.24 (0.35-4.35) | 1.71 (0.25-11.63) | 0.80 (0.30-2.12) |
| Degree of Father (Junior high school and below) | | | | | | | | |
| High/vocational high school | 0.47 (0.13-1.73) | 0.84 (0.23-3.09) | 1.74 (0.12-24.99) | 0.69 (0.19-2.50) | 1.30 (0.55-3.09) | 1.31 (0.37-4.72) | 1.21 (0.16-9.16) | 1.42 (0.51-3.96) |
| Undergraduate | 0.58 (0.18-1.87) | 0.93 (0.28-3.07) | 3.14 (0.25-38.88) | 0.73 (0.22-2.37) | 1.88 (0.83-4.28) | 2.08 (0.63-6.86) | 1.97 (0.30-13.00) | 2.27 (0.86-5.97) |
| Postgraduate | 0.53 (0.15-1.89) | 1.05 (0.30-3.61) | 1.62 (0.11-24.65) | 0.55 (0.16-1.92) | 2.41 (0.97-5.99) | 1.76 (0.51-6.12) | 1.86 (0.26-13.51) | 2.59 (0.94-7.10) |
| Premature birth (No) | | | | | | | | |
| Yes | 1.38 (0.75-2.55) | 1.54 (0.87-2.75) | 2.11 (0.68-6.56) | 0.95 (0.53-1.72) | 1.07 (0.72-1.60) | 1.01 (0.60-1.69) | **2.93 (1.23-7.00)** | 1.35 (0.86-2.12) |
| Birth mode (Vaginal delivery) | | | | | | | | |
| Cesarean section | 0.73 (0.49-1.09) | 1.30 (1.00-1.70) | 0.70 (0.34-1.45) | 0.93 (0.67-1.28) | **0.74 (0.56-0.99)** | 0.93 (0.71-1.21) | 0.81 (0.51-1.30) | 0.98 (0.78-1.22) |
| Has the child ever had pneumonia (No) | | | | | | | | |
| Yes | **2.17 (1.24-3.79)** | **1.57 (1.20-2.06)** | **4.40 (1.85-10.46)** | **3.74 (2.69-5.19)** | 1.18 (0.75-1.86) | 1.05 (0.80-1.39) | 1.16 (0.62-2.19) | **1.64 (1.30-2.07)** |
| Has the child ever had Pediatric inflammation (No) | | | | | | | | |
| Yes | 2.29 (0.76-6.86) | 1.10 (0.73-1.68) | 1.82 (0.20-16.74) | 1.12 (0.68-1.82) | **3.44 (1.38-8.59)** | 1.36 (0.90-2.05) | 2.25 (0.60-8.36) | 1.34 (0.94-1.92) |
| Has the child ever received antibiotic treatment (No) | | | | | | | | |
| Yes | 1.25 (0.77-2.04) | 1.29 (0.89-1.87) | **2.48 (1.01-6.13)** | **2.30 (1.28-4.10)** | 1.20 (0.84-1.71) | 1.28 (0.89-1.83) | **2.03 (1.18-3.49)** | **1.49 (1.09-2.04)** |
| Family historyof allergy (No) | | | | | | | | |
| Yes | **2.01 (1.32-3.07)** | **2.44 (1.83-3.25)** | 1.82 (0.82-4.01) | **2.19 (1.53-3.13)** | **2.06 (1.53-2.79)** | **2.70 (2.01-3.63)** | **3.20 (1.89-5.44)** | **2.79 (2.19-3.55)** |
| Fresh air systems use (No) | | | | | | | | |
| Sometimes | 0.78 (0.38-1.59) | 0.87 (0.56-1.36) | **3.07 (1.08-8.77)** | 0.89 (0.51-1.55) | 0.78 (0.46-1.31) | 0.87 (0.55-1.38) | 0.88 (0.36-2.12) | 0.68 (0.46-1.01) |
| Often | 0.98 (0.46-2.11) | 1.22 (0.67-2.22) | 0.31 (0.04-2.20) | **0.33 (0.11-0.98)** | 0.65 (0.35-1.21) | 0.68 (0.33-1.38) | 1.41 (0.60-3.33) | 0.64 (0.36-1.14) |
| Incense/air fresheners use (No) | | | | | | | | |
| Sometimes | 1.03 (0.50-2.12) | 0.86 (0.61-1.22) | 0.40 (0.10-1.59) | 1.06 (0.69-1.62) | 1.22 (0.72-2.07) | 0.79 (0.55-1.14) | 1.47 (0.68-3.18) | 1.06 (0.79-1.42) |
| Often | 0.66 (0.14-3.16) | 1.06 (0.51-2.20) | 0.19 (0.00-2.28) | 0.93 (0.37-2.34) | 0.84 (0.30-2.36) | 0.85 (0.40-1.79) | 3.03 (0.92-10.02) | 1.72 (0.95-3.13) |
| Open the Windows for ventilation within one day (0 times) | | | | | | | | |
| 1 | 0.33 (0.08-1.42) | 0.94 (0.08-10.45) | **0.06 (0.01-0.37)** | 0.75 (0.06-9.77) | 0.46 (0.14-1.54) | 0.25 (0.03-2.08) | **0.09 (0.02-0.49)** | 1.27 (0.12-13.15) |
| 2 | 0.39 (0.09-1.66) | 1.26 (0.11-14.06) | **0.03 (0.00-0.22)** | 0.76 (0.06-9.97) | 0.35 (0.10-1.15) | 0.34 (0.04-2.83) | 0.22 (0.05-1.09) | 1.45 (0.14-15.00) |
| ≥3 | 0.38 (0.09-1.62) | 1.02 (0.09-11.22) | **0.05 (0.01-0.29)** | 0.41 (0.03-5.37) | 0.40 (0.12-1.32) | 0.30 (0.04-2.44) | **0.19 (0.04-0.91)** | 1.44 (0.14-14.76) |
| Daily exercise time for children (< 2 hours) | | | | | | | | |
| ≥2h | 1.25 (0.81-1.92) | 1.00 (0.76-1.30) | 0.67 (0.30-1.48) | 0.89 (0.64-1.24) | 0.87 (0.63-1.20) | 1.07 (0.82-1.40) | 0.87 (0.53-1.43) | **0.77 (0.61-0.96)** |
| Daily sleep time of children (< 10 hours) | | | | | | | | |
| 10-12 h | 0.86 (0.52-1.43) | 0.98 (0.73-1.32) | 0.67 (0.28-1.64) | 1.06 (0.74-1.51) | 1.46 (0.96-2.21) | 1.04 (0.78-1.40) | 0.82 (0.47-1.41) | 1.20 (0.93-1.54) |
| ≥12 h | **0.50 (0.30-0.86)** | 1.06 (0.42-2.64) | 0.48 (0.18-1.26) | 0.94 (0.29-3.06) | 1.25 (0.82-1.89) | 0.83 (0.31-2.21) | **0.25 (0.13-0.48)** | 0.79 (0.33-1.89) |
| Residential floor (1-3 floors) | | | | | | | | |
| 4-6 floors | 0.83 (0.50-1.37) | 1.10 (0.81-1.49) | 0.40 (0.15-1.06) | 1.03 (0.71-1.49) | 1.26 (0.89-1.80) | 1.23 (0.90-1.69) | 1.59 (0.91-2.78) | 1.11 (0.85-1.45) |
| ≥7 floors | 1.17 (0.73-1.90) | 1.24 (0.89-1.73) | 0.72 (0.30-1.69) | 0.83 (0.55-1.26) | 1.24 (0.87-1.78) | 1.13 (0.80-1.59) | 1.00 (0.55-1.83) | 0.98 (0.73-1.30) |
| Decoration situation of the permanent residence (Never) | | | | | | | | |
| Before pregnancy | 0.75 (0.35-1.61) | **1.73 (1.06-2.82)** | 1.40 (0.42-4.69) | 0.63 (0.30-1.32) | 0.67 (0.37-1.21) | 0.67 (0.36-1.25) | 0.50 (0.18-1.38) | 1.17 (0.74-1.87) |
| during pregnancy | 1.85 (0.66-5.19) | 1.57 (0.75-3.28) | 2.49 (0.41-15.12) | 1.23 (0.47-3.18) | 0.72 (0.28-1.81) | 1.11 (0.50-2.44) | **0.09 (0.00-0.76)** | 0.70 (0.33-1.49) |
| after birth | 0.90 (0.39-2.08) | 1.14 (0.82-1.58) | 2.28 (0.66-7.81) | 1.31 (0.88-1.95) | 1.23 (0.69-2.20) | 1.25 (0.90-1.75) | 0.94 (0.40-2.21) | 1.21 (0.91-1.62) |
| Wall materials for children's rooms (Others) | | | | | | | | |
| wall painting | 1.29 (0.84-1.98) | 0.96 (0.71-1.29) | 0.98 (0.43-2.21) | 1.05 (0.72-1.51) | **1.40 (1.03-1.91)** | 1.19 (0.89-1.60) | 0.74 (0.43-1.28) | 1.14 (0.88-1.47) |
| Habit of keeping Windows open in summer (No) | | | | | | | | |
| Sometimes | 0.75 (0.43-1.32) | 1.07 (0.74-1.55) | 0.94 (0.33-2.68) | 1.14 (0.74-1.75) | 0.97 (0.63-1.48) | 0.84 (0.58-1.22) | 1.17 (0.60-2.28) | 1.01 (0.74-1.40) |
| Often | **0.45 (0.21-0.99)** | 1.17 (0.76-1.80) | 2.04 (0.61-6.85) | 0.70 (0.41-1.18) | 0.82 (0.47-1.42) | **0.61 (0.39-0.95)** | 1.62 (0.71-3.71) | 0.88 (0.61-1.27) |
| Habit of keeping Windows open in winter (No) | | | | | | | | |
| Sometimes | 1.02 (0.58-1.78) | 1.27 (0.85-1.91) | 0.45 (0.16-1.21) | 0.70 (0.43-1.15) | 1.15 (0.75-1.75) | 1.06 (0.70-1.59) | 0.64 (0.34-1.20) | 1.14 (0.81-1.61) |
| Often | 0.80 (0.33-1.96) | 1.24 (0.71-2.15) | 0.22 (0.05-1.05) | 0.62 (0.31-1.22) | 0.81 (0.44-1.50) | 1.06 (0.60-1.85) | **0.31 (0.11-0.83)** | 1.00 (0.64-1.58) |
| Mother's exposure to secondhand smoke from family members during pregnancy (No) | | | | | | | | |
| Yes | 1.30 (0.75-2.26) | **1.45 (1.02-2.06)** | 1.25 (0.48-3.26) | 0.88 (0.56-1.38) | 1.07 (0.71-1.62) | 1.27 (0.88-1.83) | 1.15 (0.59-2.24) | 1.04 (0.76-1.43) |
| Mother smoking (No) | | | | | | | | |
| Yes | 0.42 (0.07-2.52) | 0.92 (0.34-2.45) | 0.93 (0.14-6.43) | 1.85 (0.72-4.74) | 0.87 (0.25-3.02) | 0.40 (0.11-1.36) | 2.14 (0.42-10.80) | 0.64 (0.27-1.51) |

Data were presented with OR and 95% CI; AOR adjusted odds ratio

The bold value indicates statistical significance (*P* < 0.05)

**CHILDHOOD ALLERGY and ASTHMA COMPREHENSIVE SCREENING QUESTIONNAIRE**

Child's Name: _______________	Mother's/Father's Name: _______________

Date: _______________	Date of Birth: ____ /____/_____

Gender: Male [ ]	Female [ ]

1. Has your child ever experienced illness or discomfort after consuming one or more types of food?

Yes [ ]	No [ ]

If your answer is "No," please proceed to Question 6.

If "Yes," please continue with the following questions:

2. How often has your child experienced this illness or discomfort after consuming the food(s)?

Only once [ ]	2–4 times [ ]	More than 4 times [ ]

3. Has your child experienced any of the following symptoms within two hours after eating? (Check all that apply)

Itchy rash (hives) [ ]

Swelling of lips, eyes, or tongue [ ]

Difficulty breathing (coughing/hoarseness/wheezing) [ ]

Vomiting/diarrhea/abdominal pain [ ]

Stiff joints [ ]

Dizziness, headache, or fainting [ ]

None of the above [ ]

4. If yes to question 3, did these symptoms resolve within one day?

Yes [ ]	No [ ]	Don’t remember [ ]

5. Which of the following foods has your child reacted to within two hours of consumption? (Check all that apply)

Milk or dairy products [ ]	Eggs [ ]	Soy or soy products [ ]	Fish or shellfish [ ]	Nuts (including peanuts) [ ]	Wheat [ ]

Fruits [ ]	Don’t know [ ]	Other foods: _______

6. Has a doctor ever diagnosed your child with a food allergy?

Yes [ ]	No [ ]

7. Has your child experienced wheezing or whistling in the chest in the past 12 months?

Yes [ ]	No [ ]

If your answer is "No," please skip to Question 12.

8. How many episodes of wheezing or whistling has your child had in the past 12 months?

None [ ]	1–3 times [ ]	4–12 times [ ]	More than 12 times [ ]

9. In the past 12 months, how often has your child’s sleep been disturbed due to wheezing?

Never woke up due to wheezing [ ]

Less than one night per week [ ]

One or more nights per week [ ]

10. In the past 12 months, has your child had a dry cough at night unrelated to a cold or chest infection?

Yes [ ]	No [ ]

11. In the past 12 months, has wheezing ever been so severe that your child could only speak one or two words per breath?

Yes [ ]	No [ ]

12. Has a doctor ever diagnosed your child with asthma?

Yes [ ]	No [ ]

13. In the past 12 months, has your child had recurrent itchy or red rashes?

Yes [ ]	No [ ]

14. If your answer is "No," please skip to Question 19. If your answer to Question 13 was "Yes" how many times has your child experienced such rashes in the past 12 months?

Only once [ ]	2–3 times [ ]	4 or more times [ ]	Always [ ]

15. Which of the following areas have been affected by the rash? (Check all that apply)

16. At what age did the itchy rash first appear?

Under 2 years [ ]	2–4 years [ ]	5 years or older [ ]

17. In the past 12 months, has the rash completely disappeared at any time?

Yes [ ]	No [ ]

18. In the past 12 months, how often has your child had difficulty sleeping at night due to itchiness from the rash?

No impact [ ]

Less than one night per week [ ]

One or more nights per week [ ]

19. Has a doctor ever diagnosed your child with eczema?

Yes [ ]	No [ ]

20. In the past 12 months, has your child experienced sneezing, a runny or blocked nose, or mouth breathing unrelated to a cold or flu?

Yes [ ]	No [ ]

If your answer is "No," please skip to Question 24.

21. In the past 12 months, have these nasal symptoms been accompanied by itchy and watery eyes?

Yes [ ]	No [ ]

22. In the past 12 months, during which months did these nasal symptoms occur? (Check all that apply)

January [ ]	February [ ]	March [ ]	April [ ]

May [ ]	June [ ]	July [ ]	August [ ]

September [ ]	October [ ]	November [ ]	December [ ]

23. In the past 12 months, how much have these nasal symptoms affected your child’s daily activities?

No impact [ ]	Mild impact [ ]	Moderate impact [ ]	Severe impact [ ]

24. Has your child ever been diagnosed with seasonal pollen allergies?

Yes [ ]	No [ ]
